# Clinical decision support systems for maternity care: a systematic review and meta-analysis

**DOI:** 10.1016/j.eclinm.2024.102822

**Published:** 2024-09-05

**Authors:** Neil Cockburn, Cristina Osborne, Supun Withana, Amy Elsmore, Ramya Nanjappa, Matthew South, William Parry-Smith, Beck Taylor, Joht Singh Chandan, Krishnarajah Nirantharakumar

**Affiliations:** a Department of Applied Health Sciences, University of Birmingham, Birmingham, United Kingdom; b Department of Obstetrics and Gynaecology, Shrewsbury and Telford Hospitals NHS Trust, Telford, United Kingdom; c Keele University, Keele, United Kingdom; d Warwick Medical School, Warwick University, Coventry, United Kingdom; e Birmingham Health Partners, University of Birmingham, Birmingham, United Kingdom

**Keywords:** Clinical decision support, Systematic review, Maternity, Obstetrics, mHealth

## Abstract

**Background:**

The use of Clinical Decision Support Systems (CDSS) is increasing throughout healthcare and may be able to improve safety and outcomes in maternity care, but maternity care has key differences to other disciplines that complicate the use of CDSS. We aimed to identify evaluated CDSS and synthesise evidence of their impact on maternity care.

**Methods:**

We conducted a systematic review for articles published before 24th May 2024 that described i) CDSS that ii) investigated the impact of their use iii) in maternity settings. Medline, CINAHL, CENTRAL and HMIC were searched for articles relating to evaluations of CDSS in maternity settings, with forward- and backward-citation tracing conducted for included articles. Risk of bias was assessed using the Mixed Methods Assessment Tool, and CDSS were described according to the clinical problem, purpose, design, and technical environment. Quantitative results from articles reporting appropriate data were meta-analysed to estimate odds of a CDSS achieving its desired outcome using a multi-level random effects model, first by individual CDSS and then across all CDSS. PROSPERO ID: CRD42022348157.

**Findings:**

We screened 12,039 papers and included 87 articles describing 47 unique CDSS. 24 articles (28%) described randomised controlled trials, 30 (34%) described non-randomised interventional studies, 10 (11%) described mixed methods studies, 10 (11%) described qualitative studies, 7 (8%) described quantitative descriptive studies, and 7 (8%) described economic evaluations. 49 (56%) were in High-Income Countries and 38 (44%) in Low- and Middle-Income countries, with no CDSS trialled in both income categories. Meta-analysis of 35 included studies found an odds ratio for improved outcomes of 1.69 (95% confidence interval 1.24–2.30). There was substantial variation in effects, aims, CDSS types, context, study designs, and outcomes.

**Interpretation:**

Most CDSS evaluations showed improvements in outcomes, but there was heterogeneity in all aspects of design and evaluation of systems. CDSS are increasingly important in delivering healthcare, and Electronic Health Records and mHealth will increase their availability, but traditional epidemiological methods may be limited in guiding design and demonstrating effectiveness due to rapid CDSS development lifecycles and the complex systems in which they are embedded. Development methods that are attentive to context, such as Human Centred Design, will help to meet this need.

**Funding:**

None.

**Research in context**

**Evidence before this study**

Clinical Decision Support Systems (CDSS) have been used in healthcare for decades to improve medical decision making. Systematic reviews of both generalist and specialist CDSS have identified modest improvements in care using CDSS, but searching for CDSS and maternity in Medline, PROSPERO, and Cumulative Index to Nursing and Allied Health Literature (CINAHL) identified no reviews of CDSS use in maternity care, which is complicated by unique decision problems and risk management and thus requires its own evidence synthesis.

**Added value of this study**

This study identifies evaluations of CDSS in maternity care, providing the first systematic review of all evaluated CDSS and synthesising a varied evidence base. It finds that evaluated CDSS generally succeed in achieving the stated aims of CDSS, and provides a database of CDSS and their features that will aid clinicians, developers, implementers, and researchers of CDSS in maternity care in building and evaluating future CDSS.

**Implications of all the available evidence**

CDSS can make substantial contributions to maternity outcomes and care, but effective CDSS interventions in one context may not translate to other settings. CDSS are designed and deployed throughout maternal healthcare but few receive robust evaluations in healthcare practice and the breadth of designs in CDSS require a wide variety of disciplinary expertise to effectively research and deploy. Further research into the key ingredients of CDSS and robust design methods may help to support future implementations.

## Introduction

Preventing maternal mortality and morbidity is a core objective for health systems and forms a key metric for health performances, part of both Millenium Development Goals and Sustainable Development Goals.^1^^,^^2^ However, progress is faltering in both the Global North and South with the world's maternal mortality rate unchanged since 2013 after falling by 30% between 2000 and 2013.^3^ Khalil et al. report eight countries with significant rises in maternal mortality between 2000 and 2020, six of which are Low and Middle-Income Countries (LMICs) and two of which are High Income Countries (HICs).^4^ The burden of mortality is unequally distributed; in 2020, maternal mortality rate per 100,000 live births ranged from 1222.5 in South Sudan to 1.1 in Belarus.

Safe maternity care is a particular challenge to health systems. Maternity care is a safety critical speciality; even in HICs where adverse event rates are low,^5^ any adverse events can have tragic lifelong consequences for a child, mother or family.^6^^,^^7^ Conversely, maternity is a normal physiological process and so intervention should only take place where indicated to improve safety, the birthing experience, and to avoid unnecessary resource utilisation.^5^^,^^8^ Maternity care also faces decision challenges unique to healthcare in balancing risks between mother and foetus, such as in pre-eclampsia where earlier delivery treats a life threatening maternal condition, but can increase harm to a baby through preterm birth.^9^ Increased litigation rates exist in this pressured environment, and in 2018/2019 obstetrics accounted for 50% of medical litigation costs in the National Health Service (NHS).^10^ Reports into maternity care in the UK frequently highlight unsafe care, often focusing on variation in practice between settings and lack of risk assessment.^11^, ^12^, ^13^ There is therefore a need to standardise practice, support risk stratification, improve safety and inform decision making. Clinical Decision Support Systems have been proposed to achieve these goals.

Clinical Decision Support Systems (CDSS) are typically electronic tools that provide information to users that alters healthcare decision making.^14^ CDSS therefore improve care by firstly altering decisions,^15^ usually of healthcare staff although some target patients, leading to altered behaviour.^16^ CDSS that target safety of care or changes in practice can be viewed as implementation strategies, aiming to improve uptake of desired processes of care that lead to improved outcomes. For example, a CDSS may aim to increase rates of a process such as blood pressure monitoring according to guidelines, which leads to better treatment and fewer adverse outcomes such as eclampsia.^17^ Meta analyses often find modest impacts on measures of processes measures but few identify meaningful impacts on clinical outcomes,^18^^,^^19^ and there is little evidence to support relationships between specific CDSS features and the prediction of improvements in outcomes. Matching features to context may be key,^20^^,^^21^ and a USA-based study found that in a safety test of 8 Electronic Health Record (EHR) systems with built-in prescribing support deployed across 62 hospitals, variation in hospital safety was greater within systems than between systems.^22^ The maturity of these systems within hospitals, staff resources and buy-in, and hospital expertise were identified as affecting safety. Interactions between CDSS and their environment may lead to unintended consequences such as workflow interruption and alert fatigue,^23^, ^24^, ^25^ and case studies have reported lethal consequences varying from cognitive disruptions during complex tasks, to delaying essential and emergency care.^26^

A wide variety of CDSS exist which could be used to address maternal health problems.^27^ No systematic reviews of maternity CDSS have been published or registered to date. Speciality-specific or non-speciality systematic reviews of CDSS have been undertaken, which tend to exclude systems used in maternity care as they may be CDSS with multidisciplinary users or which use specialist software.^17^, ^18^, ^19^

Most maternity CDSS are developed for local or national implementation, with international tools such as the WHO's SMART guidelines requiring local adaptation to deploy.^28^ The unique nature of maternity care decisions means that specialist CDSS tailored to individual contexts are likely to be required. This study therefore aims to answer the questions “What CDSS have been deployed and evaluated in maternity settings?” and “Are CDSS able to improve maternity care?”

## Methods

This review was pre-registered to PROSPERO CRD42022348157 and written according to PRISMA guidelines.

### Information sources and search strategy

Articles from peer-reviewed journals identified via electronic database searches and citation tracing were included in this review. We performed database searches on 17th August 2022 without date restrictions and updated in Medline on May 24th 2024. We searched CINAHL, Medline, HMIC and CENTRAL using the search strategy in Supplementary File 1, which required search terms describing maternity care, such as “pregnancy”, and a health informatics resource, such as “decision support”. We performed forward and backward citation tracing in articles included for full-text review in Web of Science™ by extracting all references from papers and all articles citing the included paper. These papers were then included for screening, and this process was repeated until saturation was reached and no new papers included.

### Eligibility criteria

We included all articles investigating the impact of a CDSS in maternity care decisions. Wyatt et al. define a CDSS as a “system that uses two or more items of patient data to generate case-specific or encounter-specific advice”.^29^ CDSS can have a variety of different functions to support healthcare decision making including, but not limited to:

• Providing prognosis and risk-stratification advice.^30^
• Ensuring safety of care processes e.g. medication errors,^31^ antibiotic stewardship,^32^ guideline compliance.^33^
• Task-shifting to support new cadres of healthcare staff perform tasks.^34^
• Knowledge management and implementation of practice changes.^35^^,^^36^
• Supporting patients to understand outcomes of different courses of action and make informed decisions.^16^

We therefore included articles meeting the following three criteria:

1) Maternity care decisions could include decisions made by patients or practitioners, but must pertain to healthcare at any stage of pregnancy; we included deployments of risk calculators but excluded studies evaluating non-medical behaviour change only, such as weight loss advice, or neonatal care only.
2) An ‘impact’ study evaluating a deployed CDSS that was used to support real-world clinical decisions and could have changed practice, outcomes, and patient experiences during the study. We excluded studies such as simulations or laboratory studies of a CDSS, comparisons with expert opinion, or development and validation of risk calculators.
3) We use Wyatt's definition of a decision support system,^29^ including studies where any component of the intervention was a CDSS.

No language restrictions were used in screening.

### Procedures

Searches were exported into Endnote v20 from the different databases and deduplicated using the method by Bramer et al.^37^ A copy of this library was shared between reviewers, and recombined following screening to review discrepancies in inclusion. NC and CO independently performed a two-step process screening first titles and abstracts, and then full-text articles, for studies meeting our eligibility criteria. Discrepancies in inclusion were resolved by discussion between screeners. Adjudication by a third reviewer was available but never required.

Data were extracted from included articles into an Excel spreadsheet extraction form (Supplementary 2). Data was extracted by multiple reviewers for 30% of papers, ensuring that all reviewers cross-checked against every other reviewer and agreed finalised extractions, to ensure consistency across all reviewers. Disagreements were resolved by discussion or adjudicated by a third author if agreement could not be reached.

Where possible, quantitative results were extracted as odds ratios (OR) for binary outcomes with control groups, n (%) for binary outcomes without control groups, mean differences for continuous outcomes with control groups and mean (sd) for continuous outcomes without control groups. These items were calculated where feasible if not available in the original articles. Adjusted results from author analyses were extracted if presented as either OR and RR and compared the effect of a CDSS group to another group.

Text describing rationale and design of systems was copied from articles for content analysis to taxonomise the types of tools.

### Statistics

We used the Mixed Methods Assessment Tool (MMAT) to assess studies for risk of bias,^38^ except for economic studies which were assessed using the Drummond checklist.^39^ Studies were considered to be at low risk of bias overall if every dimension of the tool was low risk of bias.

Included results were summarised in tables and meta-analysed. Content analysis of decision problems, rationales, types of CDSS, and CDSS environment was used to systematically describe papers. We used the Wright taxonomy to categorise CDSS into 6 types: Expert Systems, Point of Care Alerts and Reminders, Workflow Support, Order Facilitators, Relevant Information Display, and Medication Dosing Support.^27^ We meta-analysed all quantitative binary outcomes available across included interventional studies, using a multilevel random effects model from the metafor R package^40^ that first estimated effects at the level of individual tools, then combined effects across all tools. We also meta-analysed by RCT and non-randomised interventional study subgroups. Heterogeneity was assessed using Cochran's Q and I^2^ statistics. Publication bias was assessed using funnel plots and Egger's test. A sensitivity analysis was conducted using studies considered at low risk of bias, with further details included in Supplementary 3.

### Role of funding source

This study was not funded by any external body.

## Results

### Study selection

**Fig. 1:**
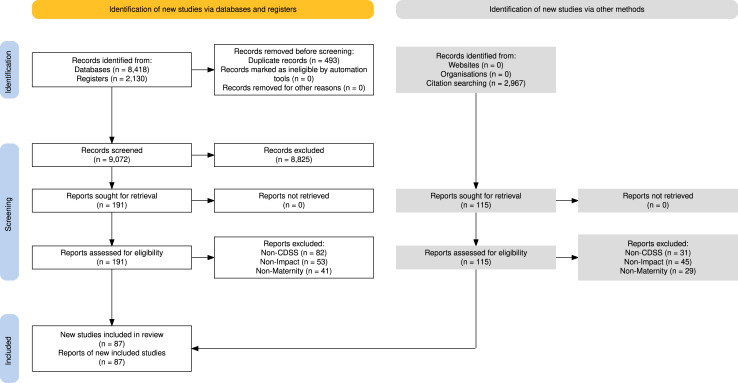
PRISMA flow diagram of article selection for this study.^41^ Period from September-2022 to May 2024 updated in medline only. CINAHL: Cumulative Index to Nursing and Allied Health Literature; CENTRAL: Cochrane Central Register of Controlled Trials; HMIC: The Health Management Information Consortium database.

The initial search query was run on 17th August 2022. 7 rounds of citation tracing are described further in Supplementary 4 and a repeat in Medline only conducted on 24th May 2024. Most records were retrieved from database and register searches (9072 unique records) and supplemented by citation tracing (2967 records) (Fig. 1).

### Study characteristics

**Fig. 2:**
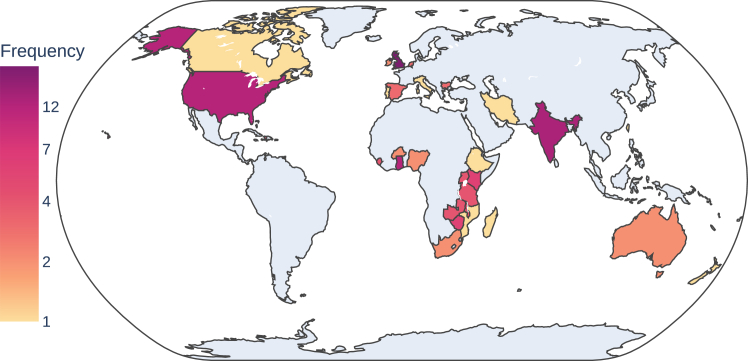
Publication of included articles on maternal Clinical Decision Support Systems by country, with colour representing the frequency of publication. Grey countries had no articles identified.

Table 1 describes characteristics of 87 reports included, of which 30 (34%) were quantitative non-randomised intervention designs and 24 (28%) were randomised controlled trials. Seven (8%) were quantitative descriptive designs, ten (11%) were qualitative and ten (11%) were mixed methods designs integrating both a qualitative and quantitative component. Seven (8%) were economic evaluations. 38 (44%) studies were conducted in LMICs, six of which took place in both Low and Lower-middle income countries. 73 studies (83%) were conducted in secondary care or tertiary settings compared with 35 (40%) in primary care settings such as community midwifery. 68 (78%) studies focused on interventions and decisions in antenatal care, with 38 (44%) investigating intrapartum decisions and only four (5%) investigating postpartum care. Fig. 2 shows the number of published articles by country, with the United Kingdom having the largest number of identified publications (19, 22%) and publications heavily concentrated in English speaking countries.

**Table 1:** Included articles and clinical decision support systems.

Article	Name	Additional description of CDSS	Decision problem	System rational	CDSS type level 1	CDSS environment	Study type	Country, year	Level of healthcare
Klumpner et al., 2020	AlertWatch OB	electronic maternal surveillance system to generate automated alerts on the labor and delivery unit based on alert criteria from he Maternal Early Warning Criteria (MEWC)	Monitoring	Trigger referral for critically ill patients; prevent missed diagnoses; improve clinical effectiveness;	Point-of-care alerts/reminders;	Software program	Qualitative	USA, April 2017–February 2019	Secondary care or higher;
Klumpner et al., 2018	AlertWatch OB	electronic maternal surveillance system to generate automated alerts on the labor and delivery unit based on alert criteria from he Maternal Early Warning Criteria (MEWC)	Monitoring	Trigger referral for critically ill patients; prevent missed diagnoses; improve clinical effectiveness;	Point-of-care alerts/reminders;	Software program	Quantitative descriptive	USA, April 2017–July 2017	Secondary care or higher;
Abasian Kasegari et al., 2020	Active labour diagnosis admission protocol	Admission protocol for women in active labour	Monitoring	standardise care; prevent missed diagnoses; Guideline adherence	Expert systems;	Paper;	RCT	Iran, September 2017–December 2017	Secondary care or higher;
Cheyne et al., 2008	Active labour diagnosis algorithm	Algorithm to improve the diagnosis of active labour in primiparous women	Monitoring	standardise care; prevent missed diagnoses; Guideline adherence	Expert systems;	Paper	RCT	UK, April 2005–June 2007	Secondary care or higher;
Usmanova et al., 2020	ASMAN	Alliance for Saving Mothers and Newborns. Provider-facing electronic platform for peripartum care including Safe Childbirth Checklist and embedded Safe Delivery App	Monitoring	Trigger referral; Guideline adherence;	Workflow Support;	Phone App;	Qualitative	India, June 2017–May 2020	Primary care; Secondary care or higher;
Usmanova et al., 2021	ASMAN	Alliance for Saving Mothers and Newborns. Provider-facing electronic platform for peripartum care including Safe Childbirth Checklist and embedded Safe Delivery App	Monitoring	Trigger referral; Guideline adherence;	Workflow Support;	Phone App;	Quantitative non-randomised	India, August 2017–March 2020	Primary care; Secondary care or higher;
Horner et al., 2013	BACIS program	The Bacis (Basic Antenatal Care Information System) Program designed to improve compliance with South African antenatal guidelines	Screening	guideline adherence; improve clinical effectiveness; standardise care	Point of care alerts/reminders; Workflow support	Software program	Quantitative non-randomised	South Africa, January 2011–June 2011	Primary care;
Luitjes et al., 2020	BIG CHANGE	BOS supported Implementation of Guidelines on Clinical Hypertension and its mANagement in GEstation trial. Computerised DSS to improve implementation of evidence-based guidelines on the management of hypertension in pregnancy	Medical conditions and prescribing	Guideline adherence; standardise care;	Expert Systems;	Software program; EHR; paper guideline	Economic evaluation	Netherlands, April 2010–May 2011	Secondary care or higher;
Luitjes et al., 2018	BIG CHANGE	BOS supported Implementation of Guidelines on Clinical Hypertension and its mANagement in GEstation trial. Computerised DSS to improve implementation of evidence-based guidelines on the management of hypertension in pregnancy	Medical conditions and prescribing	Guideline adherence; standardise care;	Expert Systems;	Software program; EHR; paper guideline	RCT	Netherlands, April 2010–May 2011	Secondary care or higher;
Abejirinde et al., 2018a	Bliss4Midwives	Diagnostic decision support for antenatal care in rural settings to reduce pregnancy-related complications by improving quality antenatal care (ANC) through non-invasive diagnostic tests supported by decision algorithms.	Screening	Trigger referral; improve safety; provide information on risks and benefits; Guideline adherence; improve clinical effectiveness	Expert systems; Relevant information display	Phone App; Software program; Device	Mixed methods	Ghana, June 2016–April 2017	Primary care; Secondary care or higher
Abejirinde et al., 2018b	Bliss4Midwives	Diagnostic decision support for antenatal care in rural settings to reduce pregnancy-related complications by improving quality antenatal care (ANC) through non-invasive diagnostic tests supported by decision algorithms.	Screening	Trigger referral; improve safety; provide information on risks and benefits; Guideline adherence; improve clinical effectiveness	Expert systems; Relevant information display	Phone App; Software program; Device	Qualitative	Ghana, June 2016–April 2017	Primary care; Secondary care or higher
Abejirinde et al., 2019	Bliss4Midwives	Diagnostic decision support for antenatal care in rural settings to reduce pregnancy-related complications by improving quality antenatal care (ANC) through non-invasive diagnostic tests supported by decision algorithms.	Screening	Trigger referral; improve safety; provide information on risks and benefits; Guideline adherence; improve clinical effectiveness	Expert systems; Relevant information display	Phone App; Software program; Device	Quantitative descriptive	Ghana, June 2016–April 2017	Primary care; Secondary care or higher
Carroll et al., 2013	CHICA	The Child Health Improvement through Computer Automation (CHICA) system. Decision support and electronic medical record system for maternal depression screening	Mental Health	improve clinical effectiveness; Trigger referral; prevent missed diagnoses;	Expert System;	EPR; Software; Paper;	RCT	USA, October 2007–July 2009	Secondary care or higher;
Long et al., 2012	Computerised Physician Order Entry system (CPOE)	Computerised physician order entry system (CPOE) Taiwan	Medical conditions and prescribing	Aid external information acquisition; improve safety	Point of care alerts/reminders	EHR	Quantitative descriptive	Taiwan, 1-year period in 2006	Secondary care or higher;
Vousden et al., 2019a	Cradle VSA	Device to measure hypovolaemic or septic shock and alert users to abnormalities in Zambia and Haiti	Monitoring	Trigger referral for critically ill patients; improve safety; better target scarce resources	Point of care alerts/reminder; Expert System	Device	Mixed methods	Kenya, Zambia, Malawi, Haiti, Sierra Leone, Zimbabwe, Uganda, India, April 2016–November 2017	Primary care; Secondary care or higher;
Vousden et al., 2018	Cradle VSA	Device to measure hypovolaemic or septic shock and alert users to abnormalities	Monitoring	Trigger referral for critically ill patients; improve safety; better target scarce resources	Point of care alerts/reminder; Expert System	Device	Mixed methods	Zimbabwe, Ethiopia, India, November 2015–January 2016	Primary care; Secondary care or higher;
Nathan et al., 2018	Cradle VSA	Device to measure hypovolaemic or septic shock and alert users to abnormalities	Monitoring	Trigger referral for critically ill patients; improve safety; better target scarce resources	Point of care alerts/reminder; Expert System	Device	Qualitative	India, Mozambique, Nigeria, South Africa, February 2014–April 2016	Primary care; Secondary care or higher;
Vousden et al., 2019b	Cradle VSA	Device to measure hypovolaemic or septic shock and alert users to abnormalities	Monitoring	Trigger referral for critically ill patients; improve safety; better target scarce resources	Point of care alerts/reminder; Expert System	Device	RCT	Kenya, Zambia, Malawi, Haiti, Sierra Leone, Zimbabwe, Uganda, India, April 2016–November 2017	Primary care; Secondary care or higher;
Vousden et al., 2019c	Cradle VSA	Device to measure hypovolaemic or septic shock and alert users to abnormalities	Monitoring	Trigger referral for critically ill patients; improve safety; better target scarce resources	Point of care alerts/reminder; Expert System	Device	RCT	Kenya, Zambia, Malawi, Haiti, Sierra Leone, Zimbabwe, Uganda, India, April 2016–Nov 2017	Primary care; Secondary care or higher;
Giblin et al., 2021	Cradle VSA	Device to measure hypovolaemic or septic shock and alert users to abnormalities	Monitoring	Trigger referral for critically ill patients; improve safety; better target scarce resources	Point of care alerts/reminder; Expert System	Device	RCT	Kenya, Zambia, Malawi, Haiti, Sierra Leone, Zimbabwe, Uganda, India, April 2016–Nov 2017	Primary care; Secondary care or higher;
Gardosi et al., 1999	Customised fundal height charts assessing growth	Customised antenatal growth chart displaying computer-generated curves for fetal weight and fundal height	Screening	Prevent missed diagnoses; trigger referral;	Workflow support; Expert system;	Paper;	Quantitative non-randomised	UK, May 1994–March 1995	Secondary care or higher;
Montgomery et al., 2007	Diamond study decision aid	VBAC Decision Aid	Mode of delivery	Aid external information acquisition; Patient empowerment and education; standardise care; provide information on risks and benefits; improve patient satisfaction	Expert systems; Relevant information display	Software program	RCT	UK, May 2004–August 2006	Secondary care or higher;
Hollinghurst et al., 2010	Diamond study decision aid	VBAC Decision Aid	Mode of delivery	Aid external information acquisition; standardise care; provide information on risks and benefits; Patient empowerment and education	Expert systems; Relevant information display	Software program	Economic evaluation	UK, May 2004–January 2006	Secondary care or higher;
Emmett et al., 2007	Diamond study decision aid	VBAC Decision Aid	Mode of delivery	Aid external information acquisition; standardise care; provide information on risks and benefits; Patient empowerment and education	Expert systems; Relevant information display	Software program	Qualitative	UK, February 2004–April 2004	Secondary care or higher;
Rees et al., 2009	Diamond study decision aid	VBAC Decision Aid	Mode of delivery	Aid external information acquisition; standardise care; provide information on risks and benefits; Patient empowerment and education	Expert systems; Relevant information display	Software program	Qualitative	UK, February 2004–April 2004	Secondary care or higher;
Venkateswaran et al., 2022	eRegQual	Palestinian integrated antenatal CDSS	Screening	Guideline adherence; improve clinical effectiveness	Expert systems	EHR	RCT	Palestine, March 2017–June 2018	Primary care;
Relph et al., 2022	GAP protocol	Growth Assessment Protocol (GAP) is a complex antenatal intervention (which trains midwives and healthcare professionals in fetal growth assessment and the use of customized centiles) that aims to increase the rate of antenatal detection of SGA and reduce stillbirth	Screening	guideline adherence; improve safety; Standardise care	Expert systems;	Software program; Paper;	Mixed methods	UK, November 2016–February 2019	Secondary care or higher;
Iliodromiti et al., 2020	GAP protocol	Growth Assessment Protocol (GAP) is a complex antenatal intervention that aims to increase the rate of antenatal detection of SGA and reduce stillbirth	Screening	guideline adherence; improve safety; Standardise care	Expert systems;	Software program; Paper;	Quantitative non-randomised	UK, 2000–2015	Secondary care or higher;
Ravula et al., 2022	GAP protocol	Growth Assessment Protocol (GAP) is a complex antenatal intervention that aims to increase the rate of antenatal detection of SGA and reduce stillbirth	Screening	guideline adherence; improve safety; Standardise care	Expert systems;	Software program; Paper;	Quantitative non-randomised	India, 2011–2018	Secondary care or higher;
Hugh et al., 2020	GAP protocol	Growth Assessment Protocol (GAP) is a complex antenatal intervention that aims to increase the rate of antenatal detection of SGA and reduce stillbirth	Screening	guideline adherence; improve safety; Standardise care	Expert systems;	Software program; Paper;	Quantitative non-randomised	UK, 2008–2017	Secondary care or higher;
Cowan et al., 2021	GAP protocol	Growth Assessment Protocol (GAP) is a complex antenatal intervention that aims to increase the rate of antenatal detection of SGA and reduce stillbirth	Screening	guideline adherence; improve safety; Standardise care	Expert systems;	Software program; Paper;	Quantitative non-randomised	New Zealand, 2012–2017	Secondary care or higher;
Vieira et al., 2022	GAP protocol	Growth Assessment Protocol (GAP) is a complex antenatal intervention that aims to increase the rate of antenatal detection of SGA and reduce stillbirth	Screening	guideline adherence; improve safety; Standardise care	Expert systems;	Software program; Paper;	RCT	UK, November 2016–February 2019	Secondary care or higher;
Vos et al., 2017	Health Pregnancy 4 All scorecared	Healthy Pregnancy 4 All' Scorecard-based antenatal risk assessment, care pathways and interdisciplinary consultation	Screening	Improved clinical effectiveness; standardise care; trigger referral	Expert systems;	Paper	Mixed methods	Netherlands, Years not provided	Primary care;
Bartlett et al., 2021	iDeliver	Digital health tool for skilled birth attendants to support maternity care in Kenya	Monitoring	Standardise care; improve clinical effectiveness; guideline adherence	Expert System; Relevant Information display; Order facilitators;	EHR; Software program	Qualitative	Kenya, December 2018–September 2020	Primary care; Secondary care or higher;
Dinh et al., 2022	iDeliver	Digital health tool for skilled birth attendants to support maternity care in Kenya	Monitoring	standardise care; improve clinical effectiveness; guideline adherence	Expert System; Relevant Information display; Order facilitators;	EHR; Software program	Quantitative non-randomised	Kenya, December 2018–September 2022	Primary care; Secondary care or higher;
Schroeder et al., 2021	INFANT	INFANT CDSS for CTG interpretation	Monitoring	improve clinical effectiveness, improve safety	Expert systems; Point of care alerts/reminders	EHR	Economic evaluation	UK; Ireland, 2010–2013	Secondary care or higher;
Wilson et al., 2021	INFANT	INFANT CDSS for CTG interpretation	Monitoring	improve clinical effectiveness, improve safety	Expert systems; Point of care alerts/reminders	EHR	Quantitative non-randomised	Australia, 2016–2019	Secondary care or higher;
Brocklehurst et al., 2017	INFANT	INFANT CDSS for CTG interpretation	Monitoring	improve clinical effectiveness, improve safety	Expert systems; Point of care alerts/reminders	EHR	RCT	UK; Ireland, 2010–2013	Secondary care or higher;
Shah et al., 2019	ImTeCHO	Innovative Mobile-phone Technology for Community Health Operations (ImTeCHO). Mobile phone application as job aid for CHWs to increase the coverage of maternal, newborn and child health services in rural India	Multi-faceted	Patient empowerment and education; improve clinical effectiveness; standardise care; guideline adherence;	Expert system; Order facilitators; workflow support;	Phone App;	Mixed methods	India, August 2013–February 2014	Primary care;
Haberman et al., 2009	IPROB	Clinical-Decision Support for documentation compliance adherence mechanism on the documentation of the estimated fetal weight and of indications for labor induction in an Electronic Medical Record clinical-decision support system	Peripartum management	Guideline adherence; standardise care	Workflow support; point of care alerts/reminders	EHR	Quantitative non-randomised	USA	Secondary care or higher;
Valdera Simbron et al., 2021	M4	Performance of the M4 model for predicting the final outcome of pregnancies resulting from ART that show low Î^2^-hCG (LB-ART) in early gestation.	Ectopic	Better target scarce resources; prevent missed diagnoses; standardise care	Expert System	Excel	Quantitative descriptive	Spain, July 2017–July 2018	Secondary care or higher;
Bobdiwala et al., 2016	M4	Performance of the M4 model for predicting the final outcome of pregnancies resulting from ART that show low Î^2^-hCG (LB-ART) in early gestation.	Ectopic	Better target scarce resources; prevent missed diagnoses; standardise care	Expert System	Excel	Quantitative non-randomised	UK, August 2012–December 2013	Secondary care or higher;
McNabb et al., 2015	m4Change App	mHeaLTH mobile phone app-based decision support and data collection app intervention for ANC in Nigeria with 4 app modules; client registration, client follow up, lab/examination and health counselling messages.	Screening	Improve patient satisfaction; guideline adherence;	point of care alerts/reminders; Order facilitators; Relevant information display;	Phone App;	Quantitative non-randomised	Nigeria, December 2012–December 2013	Primary care;
Bobdiwala et al., 2020	M6	M6 Triage of Pregnancy of Unknown Location	Ectopic	Better target scarce resources; prevent missed diagnoses; standardise care	Expert Systems	Excel	Quantitative non-randomised	UK, January 2015–January 2017	Secondary care or higher;
Blumenthal et al., 2021	MEWT	Maternity Early Warning Trigger	Monitoring	Trigger referral for critically ill patients, improve clinical outcomes	Expert system	EHR	Quantitative non-randomised	USA, year not provided	Secondary care or higher;
Shields et al., 2016	MEWT	Maternity Early Warning Trigger	Monitoring	Trigger referral for critically ill patients, improve clinical outcomes; prevent missed diagnosis	Expert system	EHR	Quantitative non-randomised	USA, January 2012–October 2015	Secondary care or higher;
Amoakoh et al., 2018	mCDMSi	mHealth clinical decision-making support intervention (mCDMSi). mHealth intervention with 4 elements: phone calls, text messaging, access to the internet, access to an unstructured supplementary service data in Eastern Region of Ghana	Multi-faceted	Guideline adherence; Improve clinical effectivess; Standardise care	Expert systems	SMS	Mixed methods	Ghana, August 2015–January 2017	Primary care; Secondary care or higher;
Amoakoh et al., 2019a	mCDMSi	mHealth clinical decision-making support intervention (mCDMSi). mHealth intervention with 4 elements: phone calls, text messaging, access to the internet, access to an unstructured supplementary service data in Eastern Region of Ghana	Multi-faceted	Guideline adherence; Improve clinical effectivess; Standardise care	Expert systems	SMS	Quantitative descriptive	Ghana, August 2015–January 2017	Primary care; Secondary care or higher;
Amoakoh et al., 2020	mCDMSi	mHealth clinical decision-making support intervention (mCDMSi). mHealth intervention with 4 elements: phone calls, text messaging, access to the internet, access to an unstructured supplementary service data in Eastern Region of Ghana	Multi-faceted	Guideline adherence; Improve clinical effectivess; Standardise care	Expert systems	SMS	Quantitative non-randomised	Ghana, August 2015–January 2017	Primary care; Secondary care or higher;
Amoakoh et al., 2019b	mCDMSi	mHealth clinical decision-making support intervention (mCDMSi). mHealth intervention with 4 elements: phone calls, text messaging, access to the internet, access to an unstructured supplementary service data in Eastern Region of Ghana	Multi-faceted	Guideline adherence; Improve clinical effectivess; Standardise care	Expert systems	SMS	RCT	Ghana, August 2015–January 2017	Primary care; Secondary care or higher;
Mackintosh et al., 2014	MOEWS	Modified Obstetric Early Warning System (MOEWS) in managing maternal complications in the peripartum period	Monitoring	Trigger referral, optimise selected patients for theatre, Trigger referral for critically ill patients	Expert system; Point of care alerts/reminders	Paper	Qualitative	UK, February 2010–August 2010	Secondary care or higher
Merriel et al., 2016	MOEWS	Modified Early Obstetric Warning System (MEOWS) in managing maternal complications in the peripartum period	Monitoring	Trigger referral, better target scarce resources, optimise selected patients for theatre, Trigger referral for critically ill patients	Expert system; Point of care alerts/reminders	Paper	Quantitative non-randomised	Zimbabwe, 2013	Primary care; Secondary care or higher;
Sheikh et al., 2017	NEWS score	NEWs tool for the management of obstetrics patients in off-work hours.	Monitoring	Trigger referral for critically ill patients, improve clinical outcomes; prevent missed diagnosis; improve safety;	Expert systems;	Paper	Quantitative non-randomised	India, September 2013–August 2014	Primary care; Secondary care or higher;
Trick et al., 2010	postpartum dTAP vaccination ordering	Computer-based clinical decision-support algorithm to increase Tdap vaccine to postpartum women according to Advisory Committee of Immunization Practices guidelines	Preventative care	Guideline adherence; standardise care	Order facilitators;	EHR	Quantitative non-randomised	USA, January 2009–April 2009	Secondary care or higher;
Benski et al., 2017	PANDA	The PANDA (Pregnancy And Newborn Diagnosis Assessment) system supports community healthcare workers in delivering antenatal care. 1. PANDA Phone: Android application to collect patient information including medical and obstetric history, to support clinical screening and provide a guide for dispensing health education. 2. PANDA point of care: biometric and pathology testing. 3. PANDA medical unit: a hospital record, allows doctors to review patients	Screening	standardise care; improve clinical effectiveness; Patient empowerment and education	Expert system	Phone App;	Quantitative descriptive	Madagascar, January 2015–March 2015	Primary care;
Kuppermann et al., 2009	Prenatal genetic testing tool	Computerized Interactive Prenatal Genetic Testing Decision-Assisting Tool in USA	Screening	Patient empowerment and education; improve patient satisfaction; provide information on risks and benefits	Expert Systems;	Software program;	RCT	USA, April 2001–April 2003	Secondary care or higher;
Carlson et al., 2019	Prenatal genetic testing tool	Computerized Interactive Prenatal Genetic Testing Decision-Assisting Tool in USA	Screening	Patient empowerment and education; improve patient satisfaction; provide information on risks and benefits	Expert Systems;	Software program;	RCT	USA, January 2017–October 2017	Secondary care or higher;
Lopes-Pereira et al., 2018	Omniview-SisPorto program	Omniview-SisPorto program- continuous cardiotocographic monitoring during labor with computer analysis and real-time alerts in UK	Monitoring	prevent missed diagnoses; Trigger referral for critically ill patients; standardise care; improve clinical effectiveness	Expert systems;	Software program; EHR; paper guideline	Quantitative non-randomised	Portugal, January 2001–December 2014	Secondary care or higher;
Ignatov et al., 2016	qCTG	quantitative Cardiotocogram management program and algorithm in a Bulgarian Hospital	Monitoring	standardise care; improve clinical effectiveness	Expert Systems;	Software program; EHR; paper guideline	RCT	Bulgaria, 2008–2011	Secondary care or higher;
Ignatov et al., 2012	qCTG	quantitative Cardiotocogram management program and algorithm in a Bulgarian Hospital	Monitoring	standardise care; improve clinical effectiveness	Expert Systems;	Software program; EHR; paper guideline	RCT	Bulgaria, 2008–2011	Secondary care or higher;
Nunes et al., 2017	Omniview-SisPorto	Omniview-SisPorto program- continuous cardiotocographic monitoring during labor with computer analysis and real-time alerts in UK	Monitoring	prevent missed diagnoses; Trigger referral for critically ill patients; standardise care; improve clinical effectiveness	Expert systems;	Software program; EHR	RCT	UK, August 2011–July 2014	Secondary care or higher;
Saccone et al., 2021	Computerised CTG	Antepartum cardiotocography with computer analysis interfaced to 2CTG2 system	Monitoring	prevent missed diagnoses; Trigger referral for critically ill patients; standardise care; improve clinical effectiveness	Expert systems;	Software program; EHR;	RCT	Italy, March 2019–December 2019	Secondary care or higher;
Saronga et al., 2015	QUALMAT	Combined decision-support and performance-based incentives to improve reported client satisfaction with maternal health services in primary facilities through support of WHO maternity and childbirth guidelines	Monitoring	improve patient satisfaction; Guideline adherence; Standardise care; improve safety	Expert systems; Workflow support	Desktop/laptop	Economic evaluation	Tanzania, June 2009–March 2013	Primary care;
Saronga et al., 2017	QUALMAT	Combined decision-support and performance-based incentives to improve reported client satisfaction with maternal health services in primary facilities through support of WHO maternity and childbirth guidelines	Monitoring	improve patient satisfaction; Guideline adherence; Standardise care; improve safety	Expert systems; Workflow support	Desktop/laptop	Economic evaluation	Tanzania, June 2009–March 2013	Primary care;
Dalaba et al., 2014	QUALMAT	Combined decision-support and performance-based incentives to improve reported client satisfaction with maternal health services in primary facilities through support of WHO maternity and childbirth guidelines	Monitoring	improve patient satisfaction; Guideline adherence; Standardise care; improve safety	Expert systems; Workflow support	Desktop/laptop	Economic evaluation	Ghana, October 2009–April 2013	Primary care;
Dalaba et al., 2015	QUALMAT	Combined decision-support and performance-based incentives to improve reported client satisfaction with maternal health services in primary facilities through support of WHO maternity and childbirth guidelines	Monitoring	improve patient satisfaction; Guideline adherence; Standardise care; improve safety	Expert systems; Workflow support	Desktop/laptop	Economic evaluation	Ghana, October 2009–April 2013	Primary care;
Sukums et al., 2015	QUALMAT	Combined decision-support and performance-based incentives to improve reported client satisfaction with maternal health services in primary facilities through support of WHO maternity and childbirth guidelines	Monitoring	improve patient satisfaction; Guideline adherence; Standardise care; improve safety	Expert systems; Workflow support	Desktop/laptop	Mixed methods	Ghana; Tanzania, October 2011–December 2013	Primary care;
Zakane et al., 2017	QUALMAT	Combined decision-support and performance-based incentives to improve reported client satisfaction with maternal health services in primary facilities through support of WHO maternity and childbirth guidelines	Monitoring	improve patient satisfaction; Guideline adherence; Standardise care; improve safety	Expert systems; Workflow support	Desktop/laptop	Qualitative	Burkina Faso, June 2012–April 2014	Primary care;
Aninanya et al., 2021	QUALMAT	Combined decision-support and performance-based incentives to improve reported client satisfaction with maternal health services in primary facilities through support of WHO maternity and childbirth guidelines	Monitoring	improve patient satisfaction; Guideline adherence; Standardise care; improve safety	Expert systems; Workflow support	Desktop/laptop	Quantitative non-randomised	Ghana, April 2012–2014	Primary care;
Mensah et al., 2015	QUALMAT	Combined decision-support and performance-based incentives to improve reported client satisfaction with maternal health services in primary facilities through support of WHO maternity and childbirth guidelines	Monitoring	improve patient satisfaction; Guideline adherence; Standardise care; improve safety	Expert systems; Workflow support	Desktop/laptop	Quantitative non-randomised	Ghana; Tanzania, 2009–2014	Primary care;
Carlisle et al., 2021	QUiPP app	The QUIPP app is a browser based support tool to stratify women into high risk and low risk of pre-term birth	Screening	Better target scarce resources; prevent missed diagnoses; improve clinical outcomes	Expert system	app; EHR	Qualitative	UK, January–March 2019	Secondary care or higher;
Watson et al., 2021	QUiPP app	The QUIPP app is a browser based support tool to stratify women into high risk and low risk of pre-term birth	Screening	Better target scarce resources; prevent missed diagnoses; improve clinical outcomes	Expert system	app; EHR	RCT	UK, March 2018–February 2019	Secondary care or higher;
Veglia et al., 2017	Risk-stratification protocol for SGA fetuses	Risk-stratification protocol for SGA fetuses	Screening	improve safety; improve clinical effectiveness; Standardise care	Expert systems;	Paper	Quantitative non-randomised	UK, October 2014–April 2016	Secondary care or higher;
Chakravarthy et al., 2019	Saving Mothers Score	Saving Mothers Score' early obstetric warning score	Screening	Trigger referral for critically ill patients, improve clinical outcomes	Expert systems; Point of care alerts/reminders	Paper	RCT	India, 2017–2018	Secondary care or higher;
Vankan et al., 2019	SIMPLE study VBAC Decision Aid	Vaginal Birth After Caesarean Section (VBAC) decision aid using a prediction model for probability of success given VBAC trial	Mode of birth	Aid external information acquisition; Patient empowerment and education; standardise care; provide information on risks and benefits; improve patient satisfaction	Expert systems	Paper	Quantitative non-randomised	Netherlands, September 2012–September 2014	Secondary care or higher;
Albert et al., 2020	Sinedie	Gestational diabetes management in a Spanish hospital	Medical conditions and prescribing	improve clinical outcomes; better target scarce resources;	Medication dosing support; Point-of-care alerts/reminders	Phone app; SMS	Quantitative non-randomised	Spain, March 2020–May 2020	Secondary care or higher;
Cabellero et al., 2017	Sinedie	Gestational diabetes management in a Spanish hospital	Medical conditions and prescribing	improve clinical outcomes; better target scarce resources;	Medication dosing support; Point-of-care alerts/reminders	Phone app; SMS	RCT	Spain, No years given	Secondary care or higher;
McCarthy et al., 2013	OTDA	Obstetric Triage Decision Aid (OTDA). Algorithms were developed for triaging two potentially serious conditions in pregnancy: pre-eclampsia and vaginal bleeding >20 weeks gestation.	Peripartum management	standardise care; improve clinical effectiveness; improve safety	Workflow support; Expert system;	EHR	Quantitative non-randomised	USA, January 2016–June 2019	Secondary care or higher;
Kuppermann et al., 2020	TOLAC decision aid	Trial of labour after cesarean (TOLAC) decision aid in USA	Mode of birth	Patient empowerment and education; provide information on risks and benefits;	Expert systems; Relevant information display	Software program	RCT	USA, January 2016–June 2019	Secondary care or higher;
Eden et al., 2014	VBAC Decision Aid	Interactive DSS to reduce decisional conflict about birth methods in previous cesarean	Mode of birth	Aid external information acquisition; Patient empowerment and education; improve patient satisfaction; provide information on risks and benefits	Expert systems;	Software program; Desktop/laptop;	RCT	USA, 2005–2007	Secondary care or higher;
McCarthy et al., 2022	OTDA	Obstetric Triage Decision Aid (OTDA). Triage tool for midwives and nurses using 10 common pregnancy complaints to risk stratify acute pregnancy problems	Peripartum management	standardise care; improve clinical effectiveness; improve safety	Workflow support; Expert system;	EHR	Quantitative descriptive	Australia, August 2017–February 2018	Secondary care or higher;
Maiga et al., 2023	REC-Maternity	Reproductive Health and Maternity Care e-registry (REC-Maternity). Electronic platform with prenatal care, labour and delivery care, postnatal care modules providing enhanced decision support and case management capacity.	Multi-faceted	Standardise care; improve clinical effectiveness; improve patient satisfaction; Patient empowerment and education;	Expert system;	EHR	Quantitative non-randomised	Burkina Faso, November 2021	Primary care
Imo et al., 2024	Opioid Personalised Prescription Protocol	CDSS to decrease the morphine equivalents prescribed while still adequately controlling pain after cesearean delivery	Medical conditions and prescribing	improve safety; standardise care;	Medication dosing support; Order facilitators;	EHR	Quantitative non-randomised	United States, March 2021–June 2022	Secondary care or higher;
Nagraj et al., 2023	SMARThealth Pregnancy	A traffic light system to highlight issues of concern requiring immediate referral and management	Screening	Trigger referral; Improve safety;	Expert system;	Phone App;	Mixed methods	India, October 2019–December 2020	Primary care;
Wang et al., 2022	PIDA	Pregnancy in IBD Decision Aid (PIDA). Patient facing decision aid to support family planning for women with inflammatory bowel disease at a range of different stages	Medical conditions and prescribing	Patient empowerment and education	Expert System; Relevant Information display;	Website	Quantitative non-randomised	Australia, Canada, Denmark, the Netherlands	Secondary care or higher;
Humphries et al., 2023	DASH-TOP	Decision Analysis in SHared decision making for Thromboprophylaxis during Pregnancy (DASH-TOP). A shared decision-making intervention that included three components: (1) direct choice exercise; (2) preference elicitation exercises and (3) personalised decision analysis	Screening	Patient empowerment and education; provide information on risks and benefits;	Expert Systems;	Software program;	Mixed methods	Canada, Spain, November 2019–March 2021	Secondary care or higher;
Kandahari et al., 2024	Oxytocin Decision Support Checklist	Oxytocin decision support checklist to standardize dosing and management of oxytocin by providing nursing staff administering the oxytocin-specific parameters for discontinuing or decreasing the dose of oxytocin, starting resuscitative measures, and notifying the supervising provider	Monitoring	Improve clinical effectiveness; improve safety; standardise care;	Point of care alerts/reminders; medication dosing support;	Paper; EHR;	Quantitative non-randomised	United States, October 2012–February 2017	Secondary care or higher;

### Risk of bias in studies

Full risk of bias assessments are presented in Supplementary 5. Eleven out of 87 studies were assessed as low risk of bias: one RCT,^42^ five qualitative studies,^43^, ^44^, ^45^, ^46^, ^47^ five quantitative non-randomised studies.^48^, ^49^, ^50^, ^51^ All other studies (n = 76) were considered to be at risk of bias.

### Results of individual studies

87 papers described in Table 1 evaluated 47 unique CDSS.^42^, ^43^, ^44^, ^45^, ^46^, ^47^, ^48^, ^49^, ^50^, ^51^, ^52^, ^53^, ^54^, ^55^, ^56^, ^57^, ^58^, ^59^, ^60^, ^61^, ^62^, ^63^, ^64^, ^65^, ^66^, ^67^, ^68^, ^69^, ^70^, ^71^, ^72^, ^73^, ^74^, ^75^, ^76^, ^77^, ^78^, ^79^, ^80^, ^81^, ^82^, ^83^, ^84^, ^85^, ^86^, ^87^, ^88^, ^89^, ^90^, ^91^, ^92^, ^93^, ^94^, ^95^, ^96^, ^97^, ^98^, ^99^, ^100^, ^101^, ^102^, ^103^, ^104^, ^105^, ^106^, ^107^, ^108^, ^109^, ^110^, ^111^, ^112^, ^113^, ^114^, ^115^, ^116^, ^117^, ^118^, ^119^, ^120^, ^121^, ^122^, ^123^, ^124^, ^125^, ^126^, ^127^ Results for all included studies are included in Supplementary 6, and vignettes describing the evidence for a selection of ten CDSS in Supplementary 7.

### Results of syntheses

We describe features of the tools evaluated by decision problem, type of tool used, rationale for CDSS use, and the environment to which it was deployed. These features are non-unique; each tool may have multiple features of the same category, and no tools had only one feature per category. Features specific to implementations, such as time periods and country of deployment, are described in Table 1 and Supplementary 6.

**Fig. 3:**
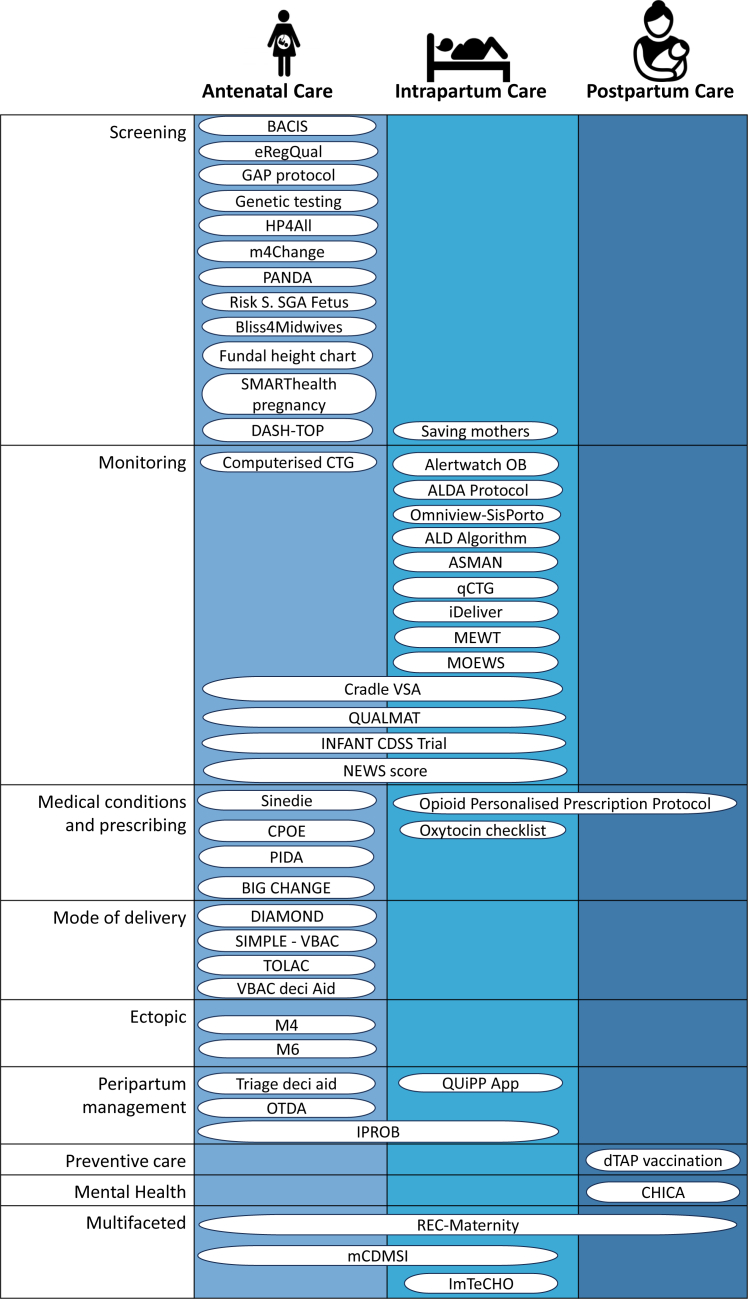
Identified tools organised by clinical problem (rows) and phase of pregnancy care (columns).

Fig. 3 shows 47 CDSS by clinical problems and the phase of care they were used in. CDSS were most commonly deployed in antenatal care (32 CDSS; 68%) on screening problems (13; 28%), such as anaemia screening which would involve a one-off blood test,^73^ and monitoring problems (14; 30%) in intrapartum care (21; 45%) such as interpreting a foetal heartbeat trace.^84^ Six (13%) systems supported medical and prescribing decisions such as whether and how to change insulin doses in patients with diabetes. Four (9%) systems supported decision-making of pregnant women to trial Vaginal Birth After C-section (VBAC). Two (4%) supported risk management in suspected ectopic pregnancies, Four (9%) support peripartum decisions such as commencing induction of labour and 1 system (2%) grouped as preventative care advised on vaccination of eligible mothers. One CDSS (2%) grouped as mental health supported screening and management of postpartum depression and three CDSS (6%) had multiple functionalities that spanned many categories. Four systems (9%) were deployed in postpartum care.

Of the 47 CDSS, Expert Systems were the most common type (37, 79%) with only 11 Point of Care Alerts and Reminders (23%), the next most common type. CDSS were intended to Standardise Care (32, 68%), Improve Clinical Outcomes (34, 72%), trigger care pathways (14, 30%), and educate and empower patients (14, 30%). They were typically deployed through Standalone Computer Programs (17, 36%), EHRs (17, 36%) or Paper (17, 32%). However, CDSS combinations of type, rationale and environment rarely reoccurred, and 37 (79%) observed combinations of CDSS types, rationales and environment were unique to that CDSS. For example, two tools (5%) were Expert Systems implemented in Excel to Standardise Care and Improve Clinical Outcomes. These tools were the M4 and M6 (a later iteration of M4) systems designed to manage pregnancy of unknown location.^49^^,^^50^ Few tools were as closely related in development, illustrating the naturally occurring variation in CDSS design.

**Fig. 4:**
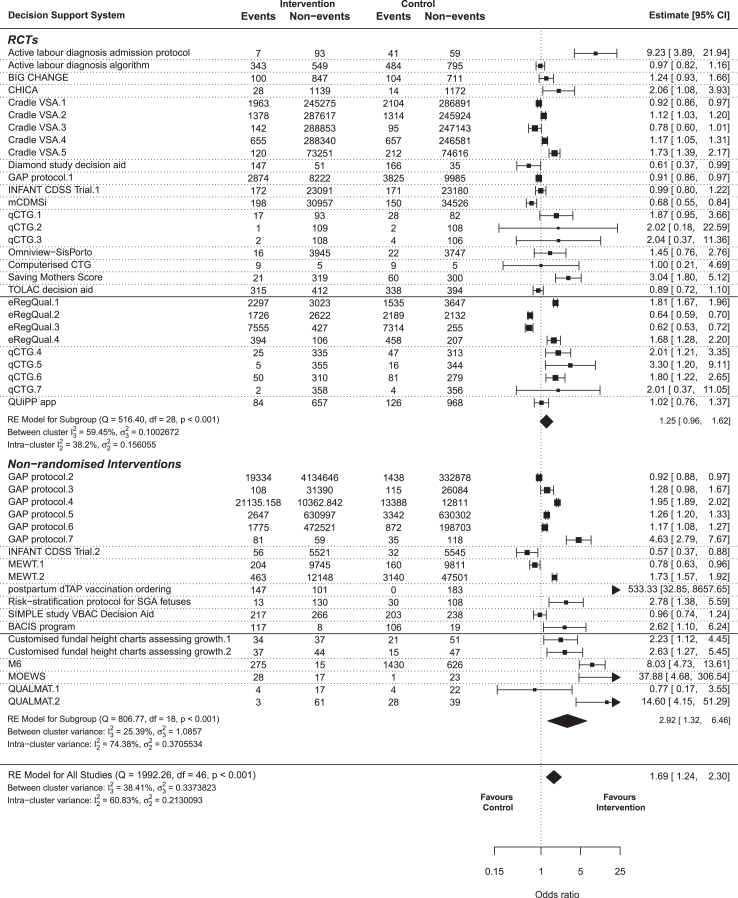
Forest plot of outcomes of interventional studies with control groups and binary outcomes. All included outcomes are displayed with odds ratio and 95% confidence interval plotted. Outcomes for each Clinical Decision Support Systems (CDSS) are combined, and then all CDSS are combined together. Dotted lines seperate CDSS, while solid lines seperate Randomised Controlled Trials (RCTs) with clinical outcomes, RCTs with process outcomes, Non-randomised interventions with clinical outcomes, and non-randomised interventions wth process outcomes. Details of outcomes analysed are provided in Supplementary 6.

Fig. 4 shows a forest plot meta-analysing results of studies of CDSS. 48 outcomes from 25 CDSS across 35 studies were included. Meta-analysis of all outcomes found that the Odds Ratio (OR) of successful outcomes that indicate better care for groups receiving CDSS was 1.69 (95% confidence interval 1.24–2.30). For a subgroup of RCT results, the OR was 1.25 (95% CI 0.96–1.62), and for non-randomised studies the OR was 2.92 (95% CI 1.24–2.30). Substantial statistical heterogeneity was observed, supporting the variation in CDSS described above. In the overall model there was greater heterogeneity within single CDSS results than between different CDSS (intra-cluster I^2^ = 60.83%, between-cluster I^2^ = 38.41%, total model I^2^ = 99.24%), suggesting that where a CDSS was evaluated for multiple outcomes or studies, there was greater variation within those results than when comparing results between CDSS. Heterogeneity remained high in the subgroup models, although between-CDSS heterogeneity was higher than within-CDSS in non-randomised studies (intra-cluster I^2^ = 74.38%, between-cluster I^2^ = 25.39%) and lower in RCTs intra-cluster I^2^ = 38.20%, between-cluster I^2^ = 59.45%. A sensitivity analysis meta-analysing results at low risk of bias found an overall OR of 1.90 (95% CI 1.08–3.33); further details are in Supplementary 3.

**Fig. 5:**
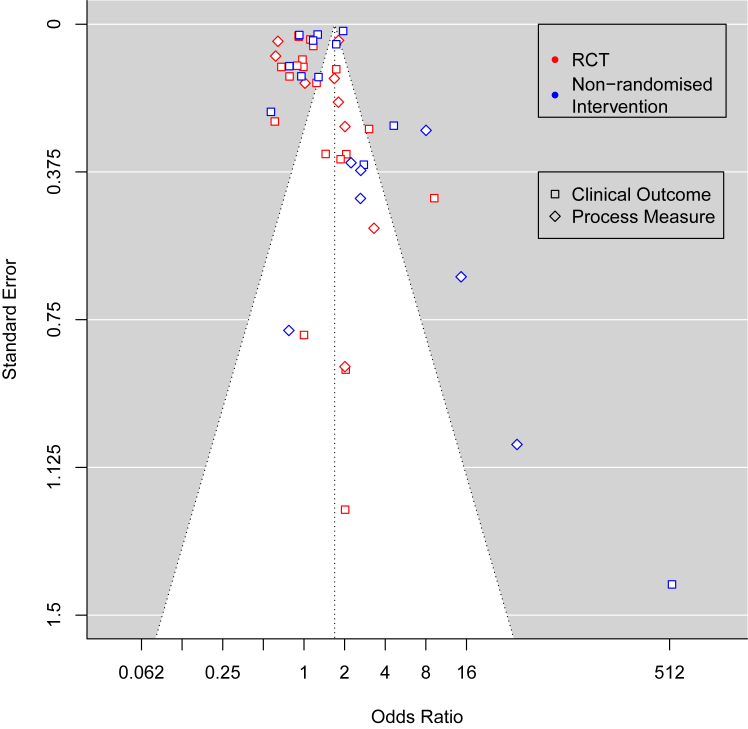
Funnel plot showing ORs against standard errors for included outcomes in the meta-analysis. The white area represents the range inside the 95% pseudo-confidence intervals.

Fig. 5 is a funnel plot showing ORs from Fig. 4 against their standard errors, and shows fewer small studies had null effect sizes than large effects. This may imply publication bias, however Egger's test for publication bias did not show evidence of missing studies (p = 0.09). When applying Egger's test to RCTs and non-randomised trials separately, this effect was further reduced (p = 0.35 for RCTs, p = 0.68 for non-randomised trials).

## Discussion

In our systematic review of clinical decision support systems, we identified 47 CDSS deployed and evaluated across 87 unique studies. CDSS designs were most commonly Expert Systems, designed for knowledge representation and user accessibility. CDSS most frequently aimed to Improve Clinical Outcomes and to Standardise Care, and were delivered through paper, standalone computer programmes, and integrated EHR environments. Meta-analysis of available quantitative outcomes found that the OR for achieving a desired outcome when using CDSS increased by 1.69 (95% confidence interval 1.24–2.30).

We identified significant variation between tools in terms of stated objectives, measured outcomes, design, and deployment context, all of which affect decisions to utilise or develop CDSS. Trick et al. demonstrated substantial effects on their desired outcome by identifying a policy with no uptake and using a workflow alteration to increase vaccine coverage from 0% to 59% of eligible post-partum mothers.^95^ The effectiveness of this relatively simple CDSS is likely driven by its contextual fit. This type of improvement is difficult to generalise due to two research challenges: identifying the active ingredients required to make improvements replicable and providing causal evidence that the CDSS resulted in the improvement. Context may be a crucial factor to understanding these active ingredients, as smaller studies in fewer centres may have better contextual fit and implementations, resulting in better outcomes than larger or randomised studies.

Numerous RCTs have investigated the causal effect of CDSS on improvements. Typically, these are cluster randomised trials, reflecting CDSS as organisational interventions with infrastructure investment and process changes that cannot be randomised to individual patients. While most trials were adequately powered to measure outcomes, many used small numbers of large clusters; the EQUIPPT trial used 11 hospitals^30^ and Cradle VSA used 10 countries,^67^ contrasting with eRegQual which used 119 antenatal clinics.^73^ Clusters are likely limited by the nature of the intervention and larger allocation samples with smaller cluster units may provide more generalisable evidence. No CDSS saw a replication trial and many did not have embedded economic analyses; CDSS trials must therefore achieve a high burden of evidence to answer their research question, as meta-analysis or further study of individual CDSS is unlikely. The INFANT trial achieved individual randomisation, however, their intervention had some important enabling features that reduced contamination between patients.^84^

Kwan et al.‘s meta-analysis of CDSS RCTs found significant increases in process outcomes (5.8% mean increase in proportion of patients receiving desired care, 95% CI 4.0, 7.6%) with no change detected in clinical outcomes (0.3% median increase in patients achieving clinical targets, 95% CI −0.7, 1.9%), smaller effects than those observed in our data and from more generalisable studies.^18^ Moja et al. observed an 22% higher risk of adverse clinical outcomes in controls, comparable to our similar RCT subgroup analysis where odds of success were 25% higher in interventions.^128^ No previous reviews have been undertaken of maternity care CDSS, but two recent neonatal CDSS reviews have been published, which have parallels with maternity care and share some intended outcomes.^129^^,^^130^ A review of features and functions of neonatal systems found the most common functionalities involved prescribing decisions, unlike in our maternal CDSS review where risk stratification was more prevalent.^130^ A systematic review focussing on CDSS predicting neonatal sepsis found consistent reductions in neonatal mortality across multiple RCTs.^129^ Single topic reviews of maternal CDSS tend to focus on patient decision aids,^16^^,^^131^ demonstrating again how maternity care CDSS differ from other specialities in that many are aimed at midwifery staff who oversee and deliver maternity care. Transferring these tools across contexts may come with additional challenges; for example, the ratio of midwives to obstetricians is over 20 times smaller in the USA than in the UK or Australia.^132^

Reviews of CDSS often focus on generalisable CDSS as therapeutic interventions, which does not adequately describe CDSS. CDSS representing guidelines are arguably implementation strategies, used to increase uptake of health interventions identified as effective.^133^, ^134^, ^135^ In contrast, risk stratification tools such as QUIPP have more in common with diagnostic or screening tools than therapies,^30^ while risk alerts/point of care monitoring systems serve as behavioural interventions targeting providers.^44^^,^^54^ While disciplines have developed around evaluating the effectiveness of these distinct approaches, evaluation becomes challenging when CDSS combine different strategies, with multidisciplinary input required to identify active ingredients of the CDSS intervention.^136^

Many CDSS evaluations measure process improvements of evidence-based care such as those identified by Kwan et al., which implies their use as implementation strategies.^18^ However, uptake of proven therapeutic interventions may not lead to improved health outcomes if the implementation strategy has unintended consequences that might prevent realisation of process benefits, such as alerts that attract attention but interrupt staff workflows in emergency situations.^26^^,^^137^ Conversely, declining CDSS use can imply its success as an implementation strategy if increases in desired care processes are sustained.

This systematic review has identified and described the broad range of evaluated CDSS tools in maternity care, offering insight into the supply and demand of decision support in the unique maternity context. As the first systematic review of maternity CDSS, we have identified a list of evaluated tools that can be used for future research and secondary analyses, and by clinicians, health services, and designers looking to utilise or develop tools for their own context. Using a snowballing methodology in addition to systematic database search identified tools that do not describe themselves as CDSS. We described the decision problems, goals, CDSS types, environments, and impact of these tools to generate insight into the key decisions involved in deploying CDSS, highlighting the evaluation challenges of identifying features of CDSS for maternity and demonstrating effectiveness.

However, this review has limitations in design. Not all CDSS are evaluated after deployment, and many developed CDSS are currently being evaluated, and therefore excluded from our review. The likely publication bias and the dominance of English-speaking countries suggests many CDSS may be absent from the published literature. The large number of tools identified through snowballing after a systematic data search suggests limitations in our search strategy. Although the strategy comprehensively identified all pre-identified CDSS, CDSS take many forms and descriptions, making truly systematic identification through traditional database searches challenging. Most CDSS that we identified were expert systems and this could be linked to our use of the Wyatt definition, as these systems are more likely to acquire information. The breadth and richness of the data produced is challenging to synthesise into clear messages for researchers and clinicians, and other existing taxonomies describing CDSS with greater focus on context^138^ and on goals^139^ could also have been used. However, for the purposes of this review the Wright taxonomy adequately described how CDSS were intended to function, and we described other features of CDSS to highlight the unique requirements of maternity care.

CDSS may have important contributions to make to maternity services by improving patient safety and supporting decision making of both staff and patients, and most evaluations report positive findings. However, delivering healthcare in maternity services may generate specific requirements of CDSS that are not observed in other specialities. Identifying features that predict effectiveness of CDSS across contexts may be a limited approach in complex health services as the requirements for CDSS vary enormously; problem-focussed approaches such as Human-Centered Design^140^ may be more promising in delivering effective CDSS that are able to positively influence healthcare decision making.

## Contributors

NC conceptualised and designed the study, curated data and verified the final dataset, performed formal analysis, created visualisations, and wrote the original draft.

CO curated data and verified the final dataset, performed formal analysis, created visualisations, and reviewed and edited the manuscript.

SW curated data, performed formal analysis, created visualisations, and reviewed and edited the manuscript.

AE curated data and reviewed and edited the manuscript.

RN curated data and reviewed and edited the manuscript.

MS reviewed and edited the manuscript.

WPS reviewed and edited the manuscript and provided supervision to NC.

BT reviewed and edited the manuscript and provided supervision to NC.

JSC reviewed and edited the manuscript and provided supervision to NC.

KN conceptualised and designed the study, reviewed and edited the manuscript and provided supervision to NC.

## Data sharing statement

All data reported in this paper is available in Supplementary 6. Analysis code in python and R is available on request.

## Editor note

The Lancet Group takes a neutral position with respect to territorial claims in published maps and institutional affiliations.

## Declaration of interests

WPS is a council member Royal College of Obstetricians and Gynecologists. MS and KN are directors of OpenClinical CIC, a not-for-profit organisation that seeks to promote the use of Clinical Decision Support technologies. MS owns stock and received royalties from Deontics Ltd., a Clinical Decision Support company whose products are not included in the reviewed papers. JSC received grant and contract funding from National Institute for Health and Care Research, Youth Endowment Fund, College of Policing, University of Birmingham, Birmingham City Council, Home Office (UK). BT received grant and contract funding from NIHR and UKRI/MRC. KN received grant and contract funding from NIHR, UKRI/MRC, Kennedy Trust for Rheumatology Research, Health Data Research UK, Wellcome Trust, European Regional Development Fund, Institute for Global Innovation, Boehringer Ingelheim, Action Against Macular Degeneration Charity, Midlands Neuroscience Teaching and Development Funds, South Asian Health Foundation, Vifor Pharma, College of Police, and CSL Behring, and consulting fees from BI, Sanofi, CEGEDIM and MSD.

## Appendix A Supplementary data

Supplementary 7

Supplementary 1 PRISMA
