# Supplementary material for: Clinical decision support systems for maternity care: a systematic review and meta-analysis
Source: eClinicalMedicine. 2024 Sep 5;76:102822. doi: 10.1016/j.eclinm.2024.102822 (PMC11408819; doi:10.1016/j.eclinm.2024.102822)
Supplement: Supplementary 1 [file mmc1.pdf]

Clinical decision support systems for maternity care

The Medline (Ovid) strategy, followed by its translation to CINAHL (Ebsco), Embase (Ovid), HMIC (Ovid), the Cochrane Library and PubMed.

Medline (Ovid):

1. exp informatics/ or exp dental informatics/ or exp medical informatics/ or exp nursing informatics/ or exp public health informatics/
2. clinical informatics.mp. [mp=ti, ab, hw, tn, ot, dm, mf, dv, kf, fx, dq, nm, ox, px, rx, ui, sy]
3. exp medical informatics/ or exp medical informatics applications/ or exp decision making, computer-assisted/ or exp decision support techniques/ or exp "information storage and retrieval"/ or exp information systems/ or exp clinical laboratory information systems/ or exp databases as topic/ or exp decision support systems, clinical/ or exp geographic information systems/ or exp hospital information systems/ or exp integrated advanced information management systems/ or exp knowledge bases/ or exp management information systems/ or exp medical records systems, computerized/ or exp medlars/ or exp online systems/ or exp radiology information systems/ or exp reminder systems/ or exp medical informatics computing/ or exp pattern recognition, automated/
4. clinical decision support$.mp. [mp=ti, ab, hw, tn, ot, dm, mf, dv, kf, fx, dq, nm, ox, px, rx, ui, sy]
5. exp Computers/ or exp Clinical Pharmacy Information Systems/ or exp Medical Order Entry Systems/ or physician order entry system.mp. or exp Medication Errors/ or exp Medication Systems, Hospital/
6. (physician order entry system$ or computeri$ed physician order entry system).mp. [mp=ti, ab, hw, tn, ot, dm, mf, dv, kf, fx, dq, nm, ox, px, rx, ui, sy]
7. (electronic$ adj3 guideline$).mp. [mp=ti, ab, hw, tn, ot, dm, mf, dv, kf, fx, dq, nm, ox, px, rx, ui, sy]
8. (electronic$ adj3 protocol).mp. [mp=ti, ab, hw, tn, ot, dm, mf, dv, kf, fx, dq, nm, ox, px, rx, ui, sy]
9. (computer$ adj3 guideline$).mp. [mp=ti, ab, hw, tn, ot, dm, mf, dv, kf, fx, dq, nm, ox, px, rx, ui, sy]
10. (computer$ adj3 protocol$).mp. [mp=ti, ab, hw, tn, ot, dm, mf, dv, kf, fx, dq, nm, ox, px, rx, ui, sy]
11. 1 or 2 or 3 or 4 or 5 or 6 or 7 or 8 or 9 or 10
12. maternity.mp. or exp Hospitals, Maternity/ or exp Obstetrics/ or exp Maternal Health Services/
13. exp Pregnancy Complications/ or exp Pregnancy/ or pregnancy.mp.
14. exp Pregnancy/ or exp Pregnant Woman/ or Mother/ or exp Obstetrics/ or exp Obstetric Delivery/ or Birth/ or exp Childbirth/ or Maternal Care/ or (pregnan* or gravid* or gestation* or 'pregnant wom#n' or matern* or mother* or obstetric* or (child adj3 bearing) or childbearing or parturition or childbirth or child-birth).ti,ab. or exp Pregnancy Outcome/ or exp Pregnancy Disorder/ or exp Pregnancy Complication/ or Pregnancy Outcome/
15. 12 or 13 or 14

CINAHL (Ebsco):

1. (MH "Health Informatics+") OR (MH "Informatics+") OR (MH “Medical Informatics+” OR (“MH Nursing Informatics+”)
2. TI clinical informatics OR AB clinical informatics OR MW clinical informatics OR SU clinical informatics
3. (MH “Medical Informatics+”) OR (MH “Decision Making, Computer Assisted+”) OR (MH “Decision Support Techniques+”) OR (MH “Information Retrieval+”) OR (MH “Information Storage+”) OR (MH “Information Systems+”) OR (MH “Health Information Systems+”) OR (MH “Clinical Information Systems+”) OR (MH “Nursing Information Systems+”) OR (MH “Clinical Laboratory Information Systems+”) OR (MH “Decision Support Systems, Clinical+”) OR (MH “Geographic Information systems+”) OR (MH “Hospital Information Systems+”) OR (MH “Integrated Advanced Information Systems+”) OR (MH “Knowledge Bases+”) OR (MH “Management Information Systems+”) OR (MH “Patient Record Systems+”) OR (MH “Electronic Health Records+”) OR (MH “Medlars+”) OR (MH “Online Systems+”) OR (MH “Radiology Information Systems+”) OR (MH “Reminder Systems+”)
4. TI clinical decision support* OR AB clinical decision support* OR MW clinical decision support* OR SU clinical decision support*

5. (MH “Computers and Computerization+”) OR (MH “Clinical Pharmacy Information Systems+”) OR (MH “Electronic Order Entry+”) OR (MH “Medication Errors+”) OR (MH “Medication Systems+”)
6. TI (physician order entry system* OR computeri?ed physician order entry system) OR AB (physician order entry system* OR computeri?ed physician order entry system) OR MW (physician order entry system* OR computeri?ed physician order entry system) OR SU (physician order entry system* OR computeri?ed physician order entry system)
7. TI (electronic* N3 guideline*) OR AB (electronic* N3 guideline*) OR MW (electronic* N3 guideline*) OR SU (electronic* N3 guideline*)
8. TI (electronic* N3 protocol) OR AB (electronic* N3 protocol) OR MW (electronic* N3 protocol) OR SU (electronic* N3 protocol)
9. TI (computer* N3 guideline*) OR AB (computer* N3 guideline*) OR MW (computer* N3 guideline*) OR SU (computer* N3 guideline*)
10. TI (computer* N3 protocol*) OR AB (computer* N3 protocol*) OR MW (computer* N3 protocol*) OR SU (computer* N3 protocol*)
11. 1 OR 2 OR 3 OR 4 OR 5 OR 6 OR 7 OR 8 OR 9 OR 10
12. maternity OR (MH “Hospitals, Special+”) OR (MH “Obstetrics+”) OR (MH “Maternal Health Services+”)
13. (MH “Pregnancy Complications+”) OR (MH “Pregnancy+”) OR pregnancy
14. (MH “Pregnancy+”) OR (MH “Expectant Mothers+”) OR (MH “Mothers+”) OR (MH “Obstetrics+”) OR (MH “Delivery, Obstetric+”) OR (MH “Childbirth+”) OR TI (pregnan* OR gravid* OR gestation* OR “pregnant wom?n” OR matern* OR mother* OR obstetric* OR (child N3 bearing) OR childbearing OR parturition OR childbirth OR child-birth) OR AB (pregnan* OR gravid* OR gestation* OR “pregnant wom?n” OR matern* OR mother* OR obstetric* OR (child N3 bearing) OR childbearing OR parturition OR childbirth OR child-birth) OR (MH “Maternal-Child Care+”) OR (MH “Pregnancy Outcomes+”) OR (MH “Pregnancy Complications+”)
15. 12 or 13 or 14

Embase (Ovid):

1. exp information science/ OR exp dental informatics/ OR exp medical informatics/ OR exp nursing informatics/
2. clinical informatics.ab,fx,hw,kf,ot,ti,dq.
3. exp medical informatics/ OR decision support system/ OR exp clinical decision support system/ OR exp information retrieval/ OR exp information system/ OR exp medical information system/ OR exp bedside information system/ OR exp nursing information system/ OR exp pathology information system/ OR exp laboratory information system/ OR exp data base/ OR exp clinical decision support system/ OR exp geographic information system/ OR exp hospital information system/ OR exp knowledge base/ OR exp electronic medical record system/ OR exp medline/ OR exp online system/ OR exp radiology information system/ OR exp reminder system/ OR exp pattern recognition
4. clinical decision support*.ab,fx,hw,kf,ot,ti,dq
5. computer/ OR exp medical information system/ OR exp physician order entry system/ OR exp computerized provider order entry/ OR exp medication error/ OR exp hospital organization
6. (physician order entry system* OR computeri#ed physician order entry system).ab,fx,hw,kf,ot,ti,dq.
7. (electronic* adj3 guideline*).ab,fx,hw,kf,ot,ti,dq.
8. (electronic* adj3 protocol).ab,fx,hw,kf,ot,ti,dq.
9. (computer* adj3 guideline*).ab,fx,hw,kf,ot,ti,dq.
10. (computer* adj3 protocol*).ab,fx,hw,kf,ot,ti,dq.
11. 1 OR 2 OR 3 OR 4 OR 5 OR 6 OR 7 OR 8 OR 9 OR 10
12. maternity OR exp maternity ward/ OR exp obstetrics/ OR exp maternal health service
13. exp pregnancy complication/ OR exp pregnancy OR pregnancy.mp
14. exp pregnancy/ OR exp pregnant woman/ OR mother/ OR exp obstetrics/ OR exp obstetric delivery/ OR exp birth/ OR exp childbirth/ OR exp maternal care/ OR (pregnan* OR gravid* OR gestation* OR ‘pregnant wom#n’ OR matern* OR mother* OR obstetric* OR (child adj3 bearing) OR childbearing OR parturition OR

childbirth OR child-birth).ti,ab. OR exp pregnancy outcome/ or exp pregnancy disorder OR exp pregnancy complication/ OR pregnancy outcome
15. 15. 12 or 13 or 14

HMIC (Ovid):

1. informatics/ OR exp health informatics/ OR exp medical informatics/ OR exp nursing informatics
2. clinical informatics.ti,ab,hw,ot,sh.
3. exp medical informatics/ OR exp computer aided decision making/ OR exp decision support systems/ OR exp decision making/ OR exp decision models/ OR exp information retrieval/ OR exp information storage/ OR exp information systems/ OR exp medical information systems/ OR exp nursing information systems/ OR exp database management systems/ OR exp geographical information systems/ OR exp hospital information systems/ OR exp knowledge base/ OR exp electronic medical record/ OR exp medline/ OR exp online system/ OR exp patient appointment reminder systems/ OR exp pattern recognition
4. clinical decision support*.ti,ab,hw,ot,sh.
5. computers/ OR exp drug information systems/ OR exp computerised information systems/ OR exp medication errors/ OR exp medication systems/
6. (physician order entry system* OR computeri#ed physician order entry system).ti,ab,hw,ot,sh.
7. (electronic* adj3 guideline*).ti,ab,hw,ot,sh.
8. (electronic* adj3 protocol).ti,ab,hw,ot,sh.
9. (computer* adj3 guideline*).ti,ab,hw,ot,sh.
10. (computer* adj3 protocol*).ti,ab,hw,ot,sh.
11. 1 OR 2 OR 3 OR 4 OR 5 OR 6 OR 7 OR 8 OR 9 OR 10
12. maternity OR exp maternity hospitals/ OR exp obstetrics/ OR exp maternity services/
13. exp pregnancy complications/ OR exp pregnancy/ OR pregnancy.mp
14. exp pregnancy/ OR exp pregnant women/ OR mothers/ OR exp obstetrics/ OR exp birth delivery/ OR exp childbirth/ OR maternal care/ OR (pregnan* OR gravid* OR gestation* OR 'pregnant wom#n' OR matern* OR mother* OR obstetric* OR (child adj3 bearing) OR childbearing OR parturition OR childbirth OR child-birth).ti,ab. OR exp pregnancy outcome/ OR exp pregnancy complications
15. 12 or 13 or 14

Cochrane Library (including CENTRAL)

The Cochrane Library does not support direct keying of MeSH terms; for each individual term, a separate search is conducted in the MeSH browser and then the desired term selected from the tree. Each MeSH term takes a line on its own so the resulting line numbering is different from the original strategy. Below is the exported search, which can also be viewed on the database at https://www.cochranelibrary.com/advanced-search/search-manager?search=6972602

#1 MeSH descriptor: [Informatics] explode all trees
#2 MeSH descriptor: [Dental Informatics] explode all trees
#3 MeSH descriptor: [Nursing Informatics] explode all trees
#4 MeSH descriptor: [Public Health Informatics] explode all trees
#5 (clinical informatics):ti,ab,kw (Word variations have been searched)
#6 MeSH descriptor: [Medical Informatics] explode all trees
#7 MeSH descriptor: [Medical Informatics Applications] explode all trees
#8 MeSH descriptor: [Decision Making, Computer-Assisted] explode all trees
#9 MeSH descriptor: [Decision Support Techniques] explode all trees
#10 MeSH descriptor: [Information Storage and Retrieval] explode all trees
#11 MeSH descriptor: [Information Systems] explode all trees
#12 MeSH descriptor: [Clinical Laboratory Information Systems] explode all trees
#13 MeSH descriptor: [Databases as Topic] explode all trees
#14 MeSH descriptor: [Decision Support Systems, Clinical] explode all trees

#15 MeSH descriptor: [Geographic Information Systems] explode all trees
#16 MeSH descriptor: [Hospital Information Systems] explode all trees
#17 MeSH descriptor: [Integrated Advanced Information Management Systems] explode all trees
#18 MeSH descriptor: [Knowledge Bases] explode all trees
#19 MeSH descriptor: [Management Information Systems] explode all trees
#20 MeSH descriptor: [Medical Records Systems, Computerized] explode all trees
#21 MeSH descriptor: [MEDLARS] explode all trees
#22 MeSH descriptor: [Online Systems] explode all trees
#23 MeSH descriptor: [Radiology Information Systems] explode all trees
#24 MeSH descriptor: [Reminder Systems] explode all trees
#25 MeSH descriptor: [Medical Informatics Computing] explode all trees
#26 MeSH descriptor: [Pattern Recognition, Automated] explode all trees
#27 (clinical decision support):ti,ab,kw (Word variations have been searched)
#28 MeSH descriptor: [Computers] explode all trees
#29 MeSH descriptor: [Clinical Pharmacy Information Systems] explode all trees
#30 MeSH descriptor: [Medical Order Entry Systems] explode all trees
#31 MeSH descriptor: [Medication Errors] explode all trees
#32 MeSH descriptor: [Medication Systems, Hospital] explode all trees
#33 ((physician order entry system*) OR (computeri?ed order entry system)):ti,ab,kw (Word variations have been searched)
#34 (electronic* guideline*) OR (electronic* protocol) OR (computer* guideline*) OR (computer* protocol*):ti,ab,kw (Word variations have been searched)
#35 #1 or #2 or #3 or #4 or #5 or #6 or #7 or #8 or #9 or #10 or #11 or #12 or #13 or #14 or #15 or #16 or #17 or #18 or #19 or #20 or #21 or #22 or #23 or #24 or #25 or #26 or #27 or #28 or #29 or #30 or #31 or #32 or #33 or #34
#36 (maternity):ti,ab,kw (Word variations have been searched)
#37 MeSH descriptor: [undefined] explode all trees
#38 MeSH descriptor: [Obstetrics] explode all trees
#39 MeSH descriptor: [Maternal Health Services] explode all trees
#40 MeSH descriptor: [Pregnancy Complications] explode all trees
#41 MeSH descriptor: [Pregnancy] explode all trees
#42 (pregnancy):ti,ab,kw (Word variations have been searched)
#43 MeSH descriptor: [Pregnant Women] explode all trees
#44 MeSH descriptor: [Mothers] explode all trees
#45 MeSH descriptor: [Obstetrics] explode all trees
#46 MeSH descriptor: [Delivery, Obstetric] explode all trees
#47 MeSH descriptor: [Parturition] explode all trees
#48 MeSH descriptor: [Maternal-Child Nursing] explode all trees
#49 MeSH descriptor: [Maternal-Child Health Services] explode all trees
#50 MeSH descriptor: [Pregnancy Outcome] explode all trees
#51 MeSH descriptor: [Pregnancy Complications] explode all trees
#52 (pregnan* OR gravid* OR gestation* OR ‘pregnant wom?n’ OR matern* OR mother* OR obstetric* or (child NEAR/3 bearing) or childbearing or parturition or childbirth or child-birth):ti,ab,kw (Word variations have been searched)
#53 #36 or #37 or #38 or #39 or #40 or #41 or #42 or #43 or #44 or #45 or #46 or #47 or #48 or #49 or #50 or #51 or #52

PubMed

1. (informatics[MeSH Terms]) OR (dental informatics[MeSH Terms]) OR (medical informatics[MeSH Terms]) OR (nursing informatics[MeSH Terms])

2. (clinical informatics[Title/Abstract])
3. (medical informatics[MeSH Terms]) OR (medical informatics applications[MeSH Terms]) OR (decision making, computer assisted[MeSH Terms]) OR (decision support techniques[MeSH Terms]) OR (information storage and retrieval[MeSH Terms]) OR (information systems[MeSH Terms] OR (clinical laboratory information systems[MeSH Terms]) OR (databases as topic[MeSH Terms]) OR (decision support systems, clinical[MeSH Terms]) OR (geographic information systems[MeSH Terms]) OR (hospital information systems[MeSH Terms]) OR (integrated advanced information management systems[MeSH Terms]) OR (knowledge bases[MeSH Terms]) OR (management information systems[MeSH Terms]) OR (computerized medical record systems[MeSH Terms]) OR (medlars[MeSH Terms]) OR (online systems[MeSH Terms]) OR (radiology information systems[MeSH Terms]) OR (reminder systems[MeSH Terms]) OR (computing, medical informatics[MeSH Terms]) OR (automated pattern recognition[MeSH Terms])
4. (clinical decision support*[Title/Abstract])
5. (computers[MeSH Terms]) OR (clinical pharmacy information systems[MeSH Terms]) OR (medical order entry systems[MeSH Terms]) OR (computerized physician order entry system[MeSH Terms]) OR (medication errors[MeSH Terms]) OR (medication systems, hospital[MeSH Terms])
6. (physician order entry system* OR computeri* physician order entry system[Title/Abstract])
7. (electronic* guideline*[Title/Abstract])
8. (electronic* protocol[Title/Abstract])
9. (computer* guideline[Title/Abstract])
10. (computer* protocol* [Title/Abstract])
11. #1 OR #2 OR #3 OR #4 OR #5 OR #6 OR #7 OR #8 OR #9 OR #10
12. (maternity[Title/Abstract]) OR (hospitals[MeSH Terms]) OR (obstetrics[MeSH Terms]) OR (maternal health services[MeSH Terms])
13. (pregnancy complications[MeSH Terms]) OR (pregnancy[MeSH Terms]) OR (pregnancy)
14. (pregnancy[MeSH Terms]) OR (pregnant woman[MeSH Terms]) OR (mother[MeSH Terms]) OR (obstetrics[MeSH Terms]) OR (obstetric delivery[MeSH Terms]) OR (birth[MeSH Terms]) OR (childbirth[MeSH Terms])) OR (pregnan*[Title/Abstract] OR gravid*[Title/Abstract] OR gestation*[Title/Abstract] OR "pregnant woman"[Title/Abstract] OR "pregnant women"[Title/Abstract] OR matern*[Title/Abstract] OR mother*[Title/Abstract] OR obstetric*[Title/Abstract] OR child bearing[Title/Abstract] OR childbearing[Title/Abstract] OR parturition[Title/Abstract] OR childbirth[Title/Abstract] OR child-birth[Title/Abstract]) OR (pregnancy outcome[MeSH Terms])) OR (pregnancy complications[MeSH Terms])
15. #12 OR #13 OR #14
