# Supplementary material for: Clinical decision support systems for maternity care: a systematic review and meta-analysis
Source: eClinicalMedicine. 2024 Sep 5;76:102822. doi: 10.1016/j.eclinm.2024.102822 (PMC11408819; doi:10.1016/j.eclinm.2024.102822)
Supplement: Supplementary 3 [file mmc3.docx]

A Systematic Review of Clinical Decision Support Systems for Maternity Care

Sensitivity Meta-analysis excluding studies assessed at increased risk of bias

**Methods**

All analyses are conducted identically to main analysis on a restricted group of studies. Both Randomised Controlled Trials and Non-Randomised Quantitative studies are included, assessed using the Mixed Methods Assessment Tool. The MMAT supports judgements in 5 key domains of a study and is not designed to produce a score that designates studies at low or high risk of bias. In this analysis, we included studies that were at low risk of bias in domains that we considered particularly important, while accepting low and high risk of domain bias in the other domains. The below table outlines these domains, which requirements were relaxed, and a brief justification for not requiring certain domains.

| Study Design | Risk of Bias Domain | Low risk required |
| --- | --- | --- |
| 2. RANDOMIZED CONTROLLED TRIALS | 2.1. Is randomization appropriately performed? | Yes |
|  | 2.2. Are the groups comparable at baseline? | Yes |
|  | 2.3. Are there complete outcome data? | Yes |
|  | 2.4. Are outcome assessors blinded to the intervention provided? | No – outcomes usually drawn from routinely collected data, generated by staff conducting interventions, and so frequently it is less possible or meaningful to blind outcome assessors |
|  | 2.5 Did the participants adhere to the assigned intervention? | No – adherence to interventions is often evaluated in other studies. Furthermore, in complex interventions such as in CDSS, it can be difficult to meaningfully separate adherence to CDSS use and adherence to the desired outcome (often processes of care). |
| 3. NON-RANDOMIZED STUDIES | 3.1. Are the participants representative of the target population? | Yes |
|  | 3.2. Are measurements appropriate regarding both the outcome and intervention (or exposure)? | Yes |
|  | 3.3. Are there complete outcome data? | Yes |
|  | 3.4. Are the confounders accounted for in the design and analysis? | Yes |
|  | 3.5. During the study period, is the intervention administered (or exposure occurred) as intended? | No – adherence to interventions is often evaluated in other studies. Furthermore, in complex interventions such as in CDSS, it can be difficult to meaningfully separate adherence to CDSS use and adherence to the desired outcome (often processes of care). |

**Results**

We included 18 (62% of those included in the main meta-analysis) outcomes from 11 (50%) CDSS for RCTs, and 4 (21%) outcomes from 4 (36%) CDSS in this sensitivity.


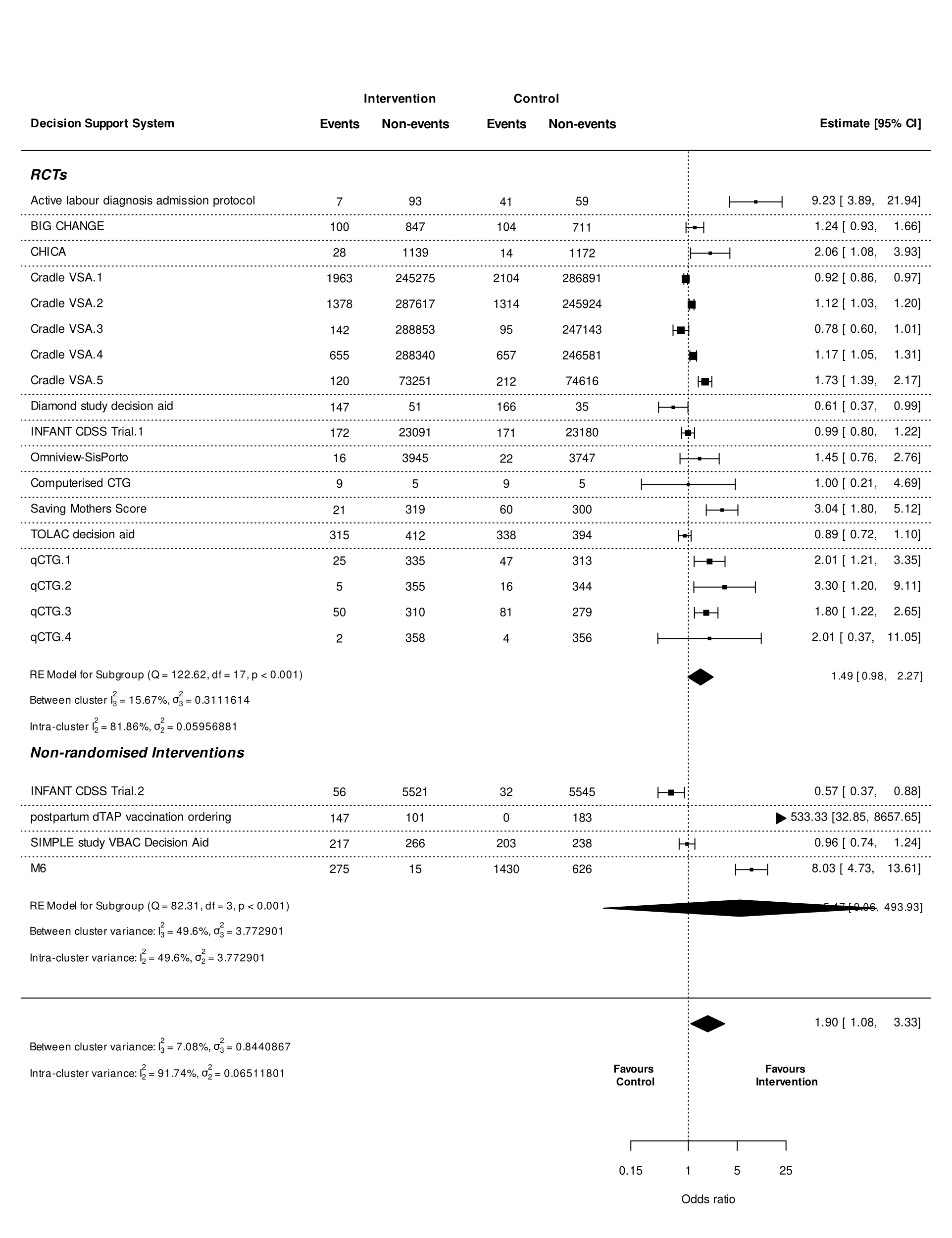


Figure 1: Subgrouped sensitivity meta-analysis forest plot.

The sensitivity analysis shows an increased effect compared to the main sensitivity analysis, due in part to the effect of a single small non-randomised interventional study with an OR of 533. The RCT subgroup however also tends towards an increased effect size. The sensitivity analysis is not statistically significantly different to the main analysis, although the precision of the results is reduced. The confidence interval of the non-randomised group is very broad, and heterogeneity is substantial.


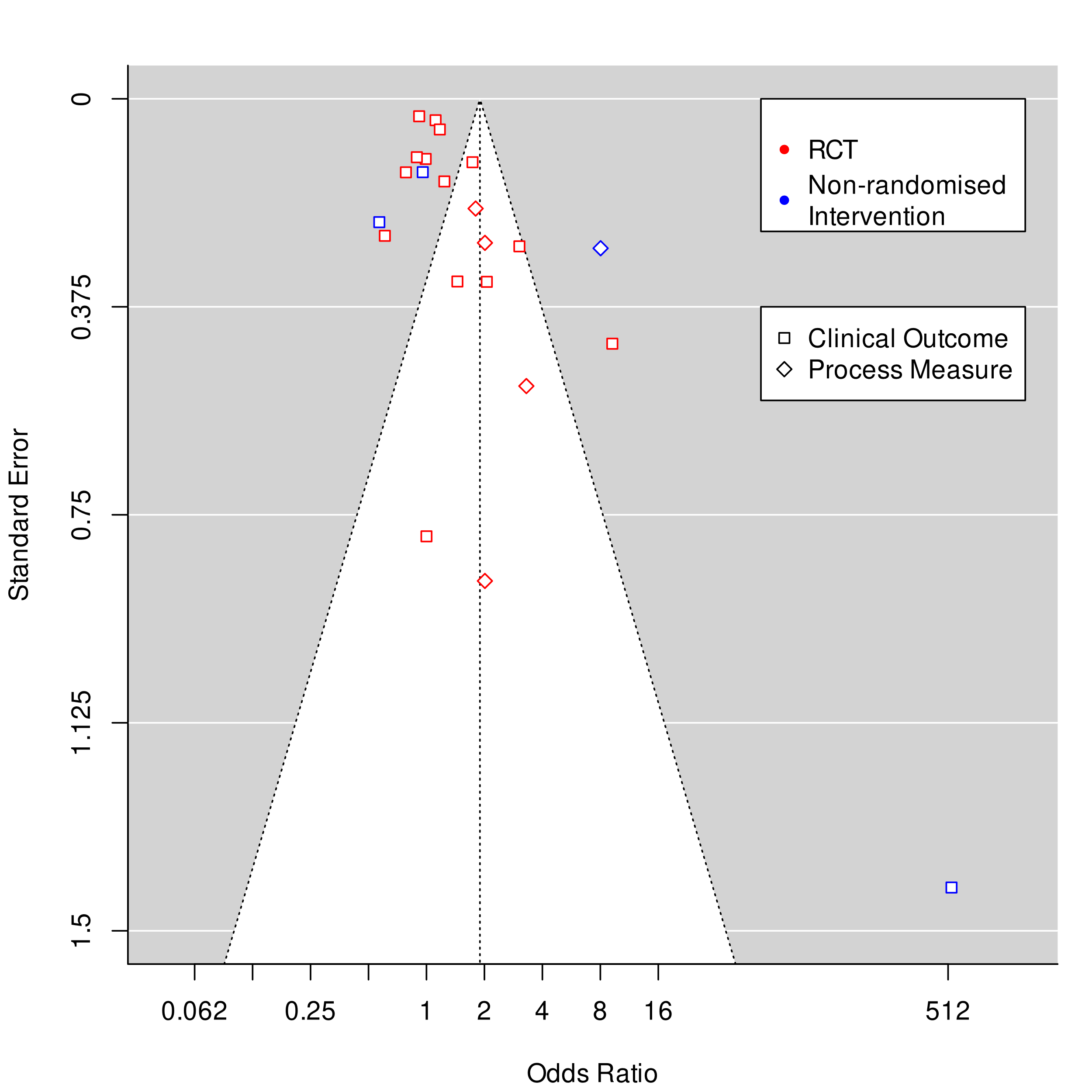


Figure 2: Sensitivity analysis funnel plot

The funnel plot shows a more centralised spread compared with the main analysis, and most results included in the sensitivity analysis have a low standard error. Egger’s test p = 0.19.
