# Supplementary material for: Clinical decision support systems for maternity care: a systematic review and meta-analysis
Source: eClinicalMedicine. 2024 Sep 5;76:102822. doi: 10.1016/j.eclinm.2024.102822 (PMC11408819; doi:10.1016/j.eclinm.2024.102822)
Supplement: Supplementary 4 [file mmc4.docx]

Supplementary 3 – Snowballing Results

Database searches retrieved 26 reports which were included in the review, while citation tracing included 55 reports by snowballing from the original 26 reports. Citation tracing occurred in 7 rounds until saturation was reached and no new records were found; the results are presented in the table below.

Table 1: Results of successive rounds of snowballing.

| Round | Date of citation tracing | New (deduplicated) records retrieved | Full text inclusion | Yield (%) |
| --- | --- | --- | --- | --- |
| 1 | 17/08/2022 | 785 | 14 | 1.7 |
| 2 | 21/10/2022 | 610 | 13 | 2.5 |
| 3 | 23/11/2022 | 352 | 12 | 3.4 |
| 4 | 02/12/2022 | 169 | 8 | 4.7 |
| 5 | 16/12/2022 | 195 | 6 | 3.1 |
| 6 | 05/01/2023 | 196 | 1 | 0.5 |
| 7 | 18/01/2023 | 35 | 0 | 0 |
