# Supplementary material for: Clinical decision support systems for maternity care: a systematic review and meta-analysis
Source: eClinicalMedicine. 2024 Sep 5;76:102822. doi: 10.1016/j.eclinm.2024.102822 (PMC11408819; doi:10.1016/j.eclinm.2024.102822)
Supplement: Supplementary 5 [file mmc5.pdf]

**Study Design**, Article	MMAT Question				
**Qualitative**	**1.1**	**1.2**	**1.3**	**1.4**	**1.5**
Carlisle 2021					
Mackintosh 2014					
Klumpner 2020					
Emmett et al. 2007					
Rees et al. 2009					
Nathan 2018					
Bartlett 2021					
Abejirinde 2018					
Zakane 2017					
Usmanova 2020					

**RCT**	**2.1**	**2.2**	**2.3**	**2.4**	**2.5**
Watson 2021					
Carlisle 2021					
Brocklehurst 2017					
Montgomery 2007					
Venkateswaran 2022					
Cabellero 2017					
Ignatov 2016					
Ignatov 2012					
Nunes 2017					
Saccone 2021					
Luitjes 2018					
Eden 2014					
Kuppermann 2009					
Carlson 2019					
Chakravarthy 2019					
Vousden 2019					
Vousden 2019					
Giblin 2021					
Amoakoh 2019					
Kupperman 2020					
Cheyne 2008					
Vieira 2022					
Abasian Kasegari, 2020					
Carroll 2010					

Quantitative non-randomised	3.1	3.2	3.3	3.4	3.5
Blumenthal 2021					
Shields 2016					
Merriel 2016					
Sheikh 2007					
Wilson 2021					
Vankan 2019					
Albert 2020					
Horner 2013					
Amoakoh 2020					
Dinh 2022					
McNabb 2015					
Trick 2010					
Iliodromiti 2020					
Ravula 2022					
Hugh 2020					
Cowan 2020					
Veglia 2017					
Aninanya 2021					
Mensah 2015					
Haberman 2009					
Usmanova 2021					
Gardosi 2005					
McCarthy et al 2013					
Bobdiwala 2020					
Bobdiwala 2016					
Lopes-Pereira 2018					
Imo 2024					
Maiga 2023					
Wang 2022					
Kandahari, 2024					

Quantitative descriptive	4.1	4.2	4.3	4.4	4.5
Klumpner 2018					
Valdera Simbron 2021					
Long 2012					
Amoakoh 2019					
Abejirinde 2019					
Benski et al. 2017					
McCarthy 2022					

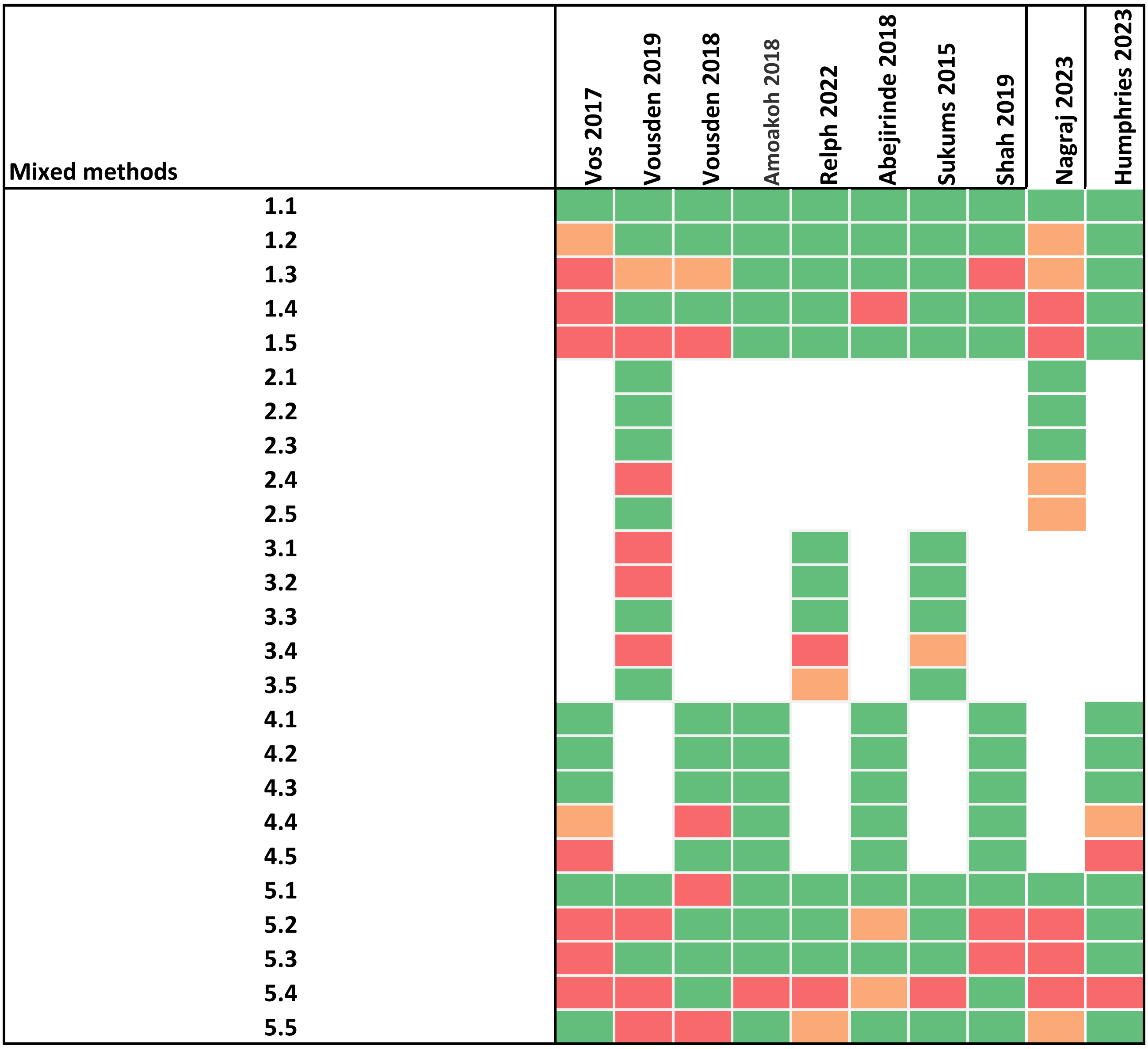
Mixed methods
Vos 2017
Vousden 2019
Vousden 2018
Amoakoh 2018
Relph 2022
Abejirinde 2018
Sukums 2015
Shah 2019
Nagraj 2023
Humphries 2023
1.1
1.2
1.3
1.4
1.5
2.1
2.2
2.3
2.4
2.5
3.1
3.2
3.3
3.4
3.5
4.1
4.2
4.3
4.4
4.5
5.1
5.2
5.3
5.4
5.5

Economic Evaluation	Schroeder, 2021	Hollinghurst, 2010	Luitjes, 2020	Saronga, 2015	Saronga, 2017	Dalaba, 2014	Dalaba, 2015
The research question is stated.							
The eco1mic importance of the research question is stated.							
The viewpoint(s) of the analysis are clearly stated and justified.							
The rationale for choosing alternative programmes or interventions compared is stated.				NA		NA	
The alternatives being compared are clearly described				NA		NA	
The form of eco1mic evaluation used is stated.							
The choice of form of eco1mic evaluation is justified in relation to the questions addressed.							
The source(s) of effectiveness estimates used are stated.							
Details of the design and results of effectiveness study are given (if based on a single study).							
Details of the methods of synthesis or meta-analysis of estimates are given (if based on a synthesis of a number of effectiveness studies).	NA	NA	NA	NA	NA	NA	NA
The primary outcome measure(s) for the eco1mic evaluation are clearly stated.							
Methods to value benefits are stated.				NA		NA	

Details of the subjects from whom valuations were obtained were given.							
Productivity changes (if included) are reported separately.	NA	NA	NA	NA		NA	NA
The relevance of productivity changes to the study question is discussed.	NA	NA	NA	NA		NA	NA
Quantities of resource use are reported separately from their unit costs.							
Methods for the estimation of quantities and unit costs are described.							
Currency and price data are recorded.							
Details of currency of price adjustments for inflation or currency conversion are given.							
Details of any model used are given.	NA	NA	NA	NA		NA	NA
The choice of model used and the key parameters on which it is based are justified.	NA	NA	NA	NA		NA	NA
Time horizon of costs and benefits is stated.						NA	
The discount rate(s) is stated.		NA	NA				
The choice of discount rate(s) is justified.		NA	NA				
An explanation is given if costs and benefits are 1t discounted.	NA				NA	NA	NA
Details of statistical tests and confidence intervals are given for stochastic data.						NA	
The approach to sensitivity analysis is given.							
The choice of variables for sensitivity analysis is justified.							
The ranges over which the variables are varied are justified.							
Relevant alternatives are compared.							
Incremental analysis is reported.	NA					NA	
Major outcomes are presented in a disaggregated as well as aggregated form.	NA						
The answer to the study question is given.							
Conclusions follow from the data reported.							
Conclusions are accompanied by the appropriate caveats.							
