# Supplementary material for: Clinical decision support systems for maternity care: a systematic review and meta-analysis
Source: eClinicalMedicine. 2024 Sep 5;76:102822. doi: 10.1016/j.eclinm.2024.102822 (PMC11408819; doi:10.1016/j.eclinm.2024.102822)
Supplement: Supplementary 7 [file mmc7.docx]

Supplementary 6 – Vignettes of evidence for selected tools

Contents

[Cradle VSA 1](#_Toc157011671)

[QUALMAT 2](#_Toc157011672)

[cGAP 2](#_Toc157011673)

[QUiPP APP 3](#_Toc157011674)

[DIAMOND 3](#_Toc157011675)

[Bliss4Midwives 4](#_Toc157011676)

[iDeliver 4](#_Toc157011677)

[Asman 5](#_Toc157011678)

[Infant 5](#_Toc157011679)

[eRegQual 5](#_Toc157011680)

### Cradle VSA

The CRADLE vital sign alert (VSA) semi-automated device measures blood pressure and pulse to calculate the pregnant mothers’ risk of hypovolaemic or septic shock, designed for use in low resource settings. The device aimed to trigger referral for critically ill patients, to improve safety, and to better target scarce resources, acting as a Point of Care alerts/reminder and Expert System. The CRADLE-VSA device was used in low and lower-middle income countries during antenatal care across all levels of care.

Six papers reported outcomes from three projects which spanned both primary and secondary or greater care settings. The CRADLE 2 study took place over 15 months (between January 2015 and April 2016) in Nigeria and South Africa. [(Nathan, 2018)](http://10.1186/s12978-017-0450-y) A feasibility study of Cradle-3 took place in Zimbabwe, Ethiopia, India from November 2015 - January 2016, and then the Cradle-3 VSA trial took place in 10 low- and middle-income country sites from April 2016-November 2017.

The qualitative evaluation of the CRADLE-2 trial (Nathan, 2018) was assessed as being of low risk of bias, while the remaining 5 out of 6 papers had high risk of bias in at least one or more MMAT components.

In the CRADLE-3 RCT there was a non-significant increase in the rate of a composite of maternal mortality or major morbidity (aOR 1.22 [95% CI 0.73-2.06]) in the intervention compared to control group (Vousden, 2019). Secondary analyses also found a non-significant increase in eclampsia (pre vs post intervention odds ratio 1.30 95% CI [0.82 2.05]) but non-significant reductions in odds of maternal death due to hypertensive disorders of pregnancy (0.84 95% CI [0.47 1.51]) [(Vousden, 2019),](http://10.1371/journal.pmed.1002775) and a statistically significant reduction in the odds of women referred for obstetric haemorrhage following intervention (0.56 [95%CI 0.42-0.74]) without any increase in bleeding-related morbidity (maternal death or emergency hysterectomy) [(Giblin, 2021).](https://bmcpregnancychildbirth.biomedcentral.com/articles/10.1186/s12884-021-03796-4)

A process evaluation during the Cradle-3 trial ranked fidelity, reach and adoption of implementing sites and found no association between these rankings and maternal morbidity and mortality (aOR 0.93; [95%CI 0.07–13.01]). [(Vousden, 2019).](http://10.1186/s13012-019-0885-3)

The CRADLE-3 feasibility study found that the intervention was perceived by women to have improved capacity to make clinical decisions around treatment and referral, escalating care and making appropriate referrals. The CRADLE-2 study similarly found that most healthcare workers perceived the CRADLE device to be easy to use and accurate. The traffic lights early warning system was unanimously reported positively, giving healthcare workers confidence with decision-making and a sense of professionalism. Unanimously, pregnant women liked the CRADLE device. The traffic light early warning system gave women and their families a better understanding of the importance of vital signs in pregnancy and during the postpartum period.

### QUALMAT

QUALMAT is standalone software that aims to improve patient satisfaction, guideline adherence, and safety as an Expert System and Workflow Support. It provides decision support based on WHO guidelines on "pregnancy, childbirth, postpartum and newborn care: a guide for essential practice”.(ref) The software was used primarily in Antenatal and Intrapartum Care in Low- and Middle-Income Countries.

Eight studies investigated the impact of QUALMAT across 3 implementations in Tanzania (Saronga 2015, 2017), Ghana (Dalaba 2014, 2015, Aninanya 2021), Burkina Faso (Zakane 2017), and studies using both Tanzanian and Ghanaian data (Sukums 2015, Mensah 2015). Aninanya alone was assessed to have low risk of bias.

Sukums (2015) evaluated uptake by looking at QUALMAT usage data compared to client registers. For ANC, 71% of clients were managed with QUALMAT in Tanzania and 59% in Ghana, and IPC, 83% in Tanzania and 67% in Ghana. In Burkina Faso, Zakane et al found that only 24% of ANC visits were managed using QUALMAT, and that QUALMAT did not fit into the workflow and features such as the partogram were not user-friendly. Mensah et al. compared the duration of ANC visits between intervention and control sites in Ghana and Tanzania finding against expectations that adjusted differences were small (additional 0.5 minute) and not statistically significant (Ghana: 0.51min [p=0.06), Tanzania: 0.54min [p=0.26]).

Aninanya et al. Constructed general satisfaction scores and evaluated the change before and after QUALMAT intervention. QUALMAT improved the general client satisfaction of ANC in the intervention site with a difference-in-differences of 0.058 (p= 0.014). The general satisfaction of the delivery outcomes were Diff-Diff of 0.072 (p=0.017).

Saronga et al. (2015) found that QUALMAT cost 52.7 USD per CDSS contact. Saronga et al. (2017) then constructed a quality score measured by questionnaire and found an increase of 4.53% in the score in implementing sites in Tanzania compared to baseline, at a economic Incremental Cost Effectiveness Ratio (ICER) of 2469 USD per percentage point of quality score for ANC and ICER of USD 338 per percentage point of quality score for childbirth. In Ghana, Dalaba et al similarly investigated average financial cost per contact using QUALMAT, at a cost per ANC contact of 4.17 USD and per birth of 26.7 USD.

### GAP

The CDSS component of GAP focuses on developing customised growth charts specific to pregnant women, delivered as part of an educational program and guideline with high-risk and low-risk pathways for managing risk of Small-for-Gestational Age (SGA). This expert system-type tool was implemented to improve safety and guideline adherence in high-income countries during antenatal care.

Our review identified 7 studies evaluating the impact of GAP in 6 implementations, all of which were assessed to have a risk of bias in at least one or more MMAT components. Initially evaluated as a paper-based customised growth charts in 1999 which showed increased odds ratio of detection of SGA of 2.23 (95% CI 1.12,4.45), 6 studies have been published since 2020 with conflicting messages on effectiveness, following greater uptake in UK hospitals from 2008. A 3-year UK-based cluster RCT of 13 sites beginning in 2016 [Vieira] showed no effect of GAP on correctly identifying SGA babies compared to standard care. Conversely, two non-randomised interventional studies both showed improvements before and after implementation; a New Zealand tertiary centre (Cowan) found increased odds ratio of detection of 4.80 (95% CI 2.82,8.18) and an Indian tertiary centre (Ravula) saw increased odds ratio of detection of 1.95 (95% CI 1.89, 2.02). In addition to this process outcome, studies also evaluated impact on stillbirths: in the same Indian study, there appeared to be a reduction in stillbirths but this was not statistically significant (Risk Ratio 0.78 [95% CI 0.60, 1.02). Two designs took ecological approaches to review the impact of GAP on national stillbirth rates: Hugh et al found that in England, stillbirths were less likely in 2017 compared to 2018, when 94 out of 133 hospital trusts had implemented GAP (OR 0.82 [95% CI 0.78, 0.86]), but a smaller and non-statistically significant effect was noted when comparing implementers to non-implementers (OR 0.94 [95% CI 0.86, 1.02]). However, Iliodromoti compared Scottish stillbirth rates, where GAP was not implemented, with England over the same period and found a greater fall in the rate of stillbirths in Scotland of 41.0 per 100,000 (95% CI 40.9,41.1).

A process evaluation embedded into the 2016 trial (ref)noted that implementation was affected by resourcing for the GAP protocol, and that changes to practice caused healthcare staff to fear they could be held accountable for negative outcomes.

### QUiPP APP

The Quantitative Innovation in Predicting Preterm birth (QUIPP) app supports identification and management of threatened preterm labour (TPTL). Its use was evaluated in a single high income country (XX), in intrapartum secondary care settings. The app was available via mobile device, webpage and some EHRs, and acted as an expert system aiming to better target scarce resources, prevent missed diagnoses and improve clinical outcomes.

3 studies evaluated QUIPP, all embedded into the EQUIPTT trial in England which was a 13-unit cluster RCT. [(Carlisle, 2021a)](http://Carlisle) and [(Carlisle, 2021b)](http://10.1016/j.midw.2020.102864) were assessed as low risk of bias.

The RCT did not provide evidence of a reduction in a composite outcome of inappropriate admission or discharge decisions of TPTL per woman (OR 0.97 [95% CI 0.66-1.42]) (Watson et al 2021). In a subset of participants from the EQUIPTT study investigating pregnant women’s experiences with the QUIPP app [(Carlisle, 2021)](http://10.1016/j.midw.2020.102864), the reduction in anxiety measured on a Visual Analogue Scale for Anxiety (VASA) scores for women aware of the QUIPP app being used compared with those not aware that the app was being used fell just below the statistical significance threshold (mean difference 0.84 [95% CI 1.758, -0.078]), and odds of women experiencing high decisional conflict about management decisions for women who were aware of the QUiPP app use showed no evidence of effect (OR 0.71 [95%CI 0.29, 1.76]).

Qualitative findings from the EQUIPTT study [(Carlisle, 2021)](https://bmcmedinformdecismak.biomedcentral.com/articles/10.1186/s12911-021-01681-w) found that clinicians appeared to find the QUiPP app an accessible and acceptable clinical that changed how they perceived TPTL risk and increased their confidence in clinical decision making. Clinicians also felt the QUiPP scores allowed them to have better conversations with women they cared for. Staff shortages, busy acute settings and hospital culture affected app implementation.

### DIAMOND

The DIAMOND trial tested an unnamed computer-based decision aid aiming to reduce decisional conflict and help pregnant women who previously had a caesarean section to decide on mode of delivery. The software program acted as an expert system and aimed to aid external information acquisition, standardise care, provide information on risks and benefits and improve patient empowerment and education. The tool was used as part of ANC in secondary care settings in a high income country.

Four studies evaluated the decision aid, and two of the four studies were assessed as having low risk of bias. The individual RCT (DIAMOND) was conducted in four maternity units in the UK from May 2004 to August 2006 (Montgomery, 2007) and tested a CDSS, an information only program and usual care. The increased rate of vaginal birth was not statistically significant for women in the CDSS group compared to usual care (37% *v* 30%, aOR 1.42, [95CI 0.94, 2.14]), and decision analysis groups had reduced mean decisional conflict scores on a 25-point scale compared with women in the usual care group (−4.0 [95%CI −6.5, −1.5]). The embedded cost-consequence economic evaluation (Hollinghurst 2010) estimated mean total cost per mother and baby with CDSS to be similar to usual care (-4.52 [95%CI –172, 107]).

Prior to the trial the tool was piloted and women’s views of the acceptability and usability of the programs investigated through semi-structured 1-1 interviews (Emmett). Overall, women found the CDSS useful, with the main contrast in women’s views concerning the two decision aids related to the difficulty some users of the decision analysis program experienced correctly completing the ratings task. Healthcare practitioner views on decision aid implementation (Rees) were tested at the end of DIAMOND trial. Overall, women found the program content and the CDSS informative and useful. The main contrast in views concerning the two decision aids related to the difficulty some users of the decision analysis program experienced correctly completing the ratings task.

### Bliss4Midwives

Bliss4Midwives (B4M) consists of a non-invasive monitoring device with integrated decision support. Decision support focussed on risk assessment of pre-eclampsia, gestational diabetes and anaemia and identifying women who required referral.

Three studies evaluated B4M, of which one (Abejerinde 2018b) was assessed to have low risk of bias. Abejerinde 2019 observed 7 health facilities implementing the device and found that of 708 women who may have required non-urgent referral for investigation, 335 (40%) were investigated and managed onsite using B4M, and 107 women were urgently referred for further management of 835 women who presented to care. A mixed methods realist review surveyed users and found no evidence of difference in usability ratings between the low-uptake and moderate-uptake clusters, and a linked qualitative study found time required to offer care increased, but women treated using B4M felt more listened to and expressed greater trust in managements plans made using the device. Health workers felt B4M may have increased demand for services at the facilities using the device.

### iDeliver

iDeliver is an integrated health record with clinical decision support and educational functionality, deployed in a Kenyan hospital in 2018. CDSS components brought together and described a range of intrapartum guidelines to improve guideline adherence and clinical effectiveness through Expert System, Relevant Information display, and Order Facilitator mechanisms.

Two studies evaluated iDeliver, of which Dinh et al may have been at low risk of bias. Dinh et al extracted usage data from the system, finding that 45% of babies who delivered at the hospital were recorded in iDeliver. Data entry for most variables improved over time (from X to Y%). CDSS components were not specifically evaluated. Bartlett et al conducted a qualitative evaluation alongside describing a detailed design process. Participants reported that the tool was easy to learn and use, and specific features were highlighted as helpful by different participants, but the additional burden of documentation and tech interaction was challenging in the low-resource environment and high workloads meant care recommendations from the tool were not completed in the available time.

### Asman

ASMAN (Alliance for Saving Mothers and Newborns) is a provider-facing mobile device app for peripartum care including a Safe Childbirth Checklist and the embedded Safe Delivery App. The CDSS acted as a workflow support system and aimed to trigger referral and improve guideline adherence. ASMAN was evaluated throughout pregnancy, in all levels of care, in a lower middle income country.

Of 2 papers evaluating ASMAN, 1 paper had a low risk of bias (Usmanova 2021). They found that data entry completeness varied substantially at different stages of pregnancy care, being lowest during at the PNC period (40.5%), followed by admission (80.2%), discharge (82.9%), post-delivery (93.1%) and delivery (93.7%). A semi-structured interview study of 44 providers found that ASMAN improved provider’s ability to take a complete history and physical exam, identify high-risk patients, manage cases confidently, facilitate provider communication, improve reporting processes, and ensure continuity of care for referral patients. Respondents also perceived improvements in peripartum outcomes and related metrics at their facilities after the introduction of ASMAN, including decreases in obstetric complications and stillbirth rates, and increases in timely referrals.

### Infant

INFANT is a decision support system for identifying foetal distress on cardiotocography monitoring. It is integrated into a maternity electronic health record used in several high-income countries, acting as both expert system and high-risk state monitoring to improve safety and clinical outcomes.

Three studies evaluated outcomes of INFANT, of which Wilson et al may have been at low risk of bias. Brocklehurst et al conducted an individually RCT from 2010-2013 in UK and Ireland maternity units and found no effect on a composite neonatal outcome including stillbirth and deaths (Risk ratio 1.01 [95%CI 0.82, 1.25]), or on developmental outcomes (PARCA-R score mean difference 0.63 [95%CI -0.98, 2.25]). Schroeder et al.’s economic evaluation of the same trial found no differences in costs between interventions and controls for mothers during admissions (22.03 [95%CI −4.79 to 48.85]) or after discharge, (−178.27 [95%CI −364.28, 7.74]). In contrast, a before-and-after Australian tertiary centre interventional study by Wilson et al. found a reduced odds ratio of a similar composite neonatal outcome of 0.57 (95%CI 0.37, 0.88).

### eRegQual

eRegQual is an integrated electronic health record with clinical decision support implemented across Palestinian antenatal clinics. Bringing together a range of clinical guidelines as an expert system, it provided diagnostic support and treatment functions to users.

A cluster RCT evaluating eRegQual, with some risk of bias due to lack of blinding in the trial, evaluated guideline adherence for management of anaemia (adjusted Odds Ratio of 1.88 [95%CI 1.52,2.32]), diabetes (aOR 1.45 [95%CI 1.14,2.05]), hypertension (aOR 1.62 [95%CI 1.29, 2.05]), and abnormal foetal growth (aOR 0.59 [95%CI 0.37,0.96]). Clinics with higher patient volumes, attached laboratories and older populations appeared to have better performance.
